# Solid stress-induced migration is mediated by GDF15 through Akt pathway activation in pancreatic cancer cells

**DOI:** 10.1038/s41598-018-37425-6

**Published:** 2019-01-30

**Authors:** Maria Kalli, Angeliki Minia, Vaia Pliaka, Christos Fotis, Leonidas G. Alexopoulos, Triantafyllos Stylianopoulos

**Affiliations:** 1 Cancer Biophysics Laboratory, Department of Mechanical and Manufacturing Engineering, University of Cyprus, Nicosia, Cyprus; 2 ProtATonce Ltd, Athens, Greece; 3 Department of Mechanical Engineering, National Technical University of Athens, Athens, Greece

## Abstract

Solid stress is a biomechanical abnormality of the tumor microenvironment that plays a crucial role in tumor progression. When it is applied to cancer cells, solid stress hinders their proliferation rate and promotes cancer cell invasion and metastatic potential. However, the underlying mechanisms of how it is implicated in cancer metastasis is not yet fully understood. Here, we used two pancreatic cancer cell lines and an established *in vitro* system to study the effect of solid stress-induced signal transduction on pancreatic cancer cell migration as well as the mechanism involved. Our results show that the migratory ability of cells increases as a direct response to solid stress. We also found that Growth Differentiation Factor 15 (GDF15) expression and secretion is strongly upregulated in pancreatic cancer cells in response to mechanical compression. Performing a phosphoprotein screening, we identified that solid stress activates the Akt/CREB1 pathway to transcriptionally regulate *GDF15* expression, which eventually promotes pancreatic cancer cell migration. Our results suggest a novel solid stress signal transduction mechanism bringing GDF15 to the centre of pancreatic tumor biology and rendering it a potential target for future anti-metastatic therapeutic innovations.

## Introduction

Solid stress - the mechanical forces per unit area generated by the solid phase of a tumor during progression - is a characteristic biomechanical abnormality of the tumor microenvironment that is rapidly gaining ground as an important regulator of cancer progression^1^. Solid stress arises from the increased mechanical forces in the tumor interior, caused by the excessive accumulation of its structural components (e.g., cancer and stromal cells and extracellular matrix) within the restricted environment of the host tissue^2,3^. It is well known that solid stress inhibits tumor growth, induces cell apoptosis and regulates tumor morphology^4–7^, while a limited number of studies has shown that solid stress can also enhance the metastatic potential of cancer cells^6,8–10^. Specifically, mechanical compression of about 6.0 mmHg has been found to promote the invasion of mammary carcinoma cells through a subset of “leader cells” that have the capacity of forming filopodia at the leading edge of the cell sheet^8^. In a more recent study, it was shown that peripheral cells growing under confined conditions within multicellular spheroids, were more proliferative and migratory, suggesting that mechanical stimuli from the surrounding microenvironment might promote cancer cell invasion^6^. Moreover, an exogenously-induced predefined mechanical compression of about 9.0 mmHg applied on colon crypts has been found to stimulate Ret/β-catenin/Myc pathway *in vivo*, contributing to the growth and spread of the tumor^9^. Recently, it has also been proposed that mechanical compression (5.0 mmHg) in combination with Interleukin-6 (IL-6) treatment activates the Akt/Gsk-3β/β-catenin signaling pathway to induce epithelial-to-mesenchymal transition (EMT) in renal cell carcinoma^11^. However, data regarding the exact mechanism by which solid stress promotes cellular responses, especially in pancreatic cancer, are limited.

In our previous work, we showed that solid stress activates normal pancreatic fibroblasts enabling them to promote pancreatic cancer cell migration through the secretion of Growth Differentiation Factor-15 (GDF15), which is strongly elevated in fibroblasts as a response to mechanical compression^12^. GDF15, which is also known as Macrophage Inhibitory Cytokine 1 (MIC-1), has been shown to be upregulated in several aggressive tumor types such as glioblastoma, pancreatic, breast and colorectal cancers and it has been correlated with poor prognosis and patient survival^13–16^. With regard to its mechanism of action, it has been proposed that GDF15 is regulated by PTEN/PI3K/Akt signaling pathway in lung cancer cells^17^, while it can promote metastasis of liver and colorectal tumors through Akt/Gsk-3β/β-catenin or Smad2/3 signaling pathways, respectively^18,19^. However, the mechanism by which GDF15 is regulated in response to mechanical cues as well as the way by which it can promote solid stress-induced pancreatic tumor progression are not yet fully defined.

In the present study, we aimed to investigate the effect of solid stress on the migration of two distinct pancreatic cancer cell lines, MIA PaCa-2 and BxPC-3, using a previously-described *in vitro* transmembrane pressure device^1,5,8,11,12,20^. Our findings led us to form the hypothesis that solid stress could be driven intracellularly by a signal transduction mechanism in order to regulate cellular responses, and particularly cell migration. We conclude that solid stress signal transduction is mediated by an Akt-dependent mechanism that eventually promotes GDF15-induced pancreatic cancer cell migration.

## Results

### Mechanical Compression promotes pancreatic cancer cell migration

**Figure 1:**
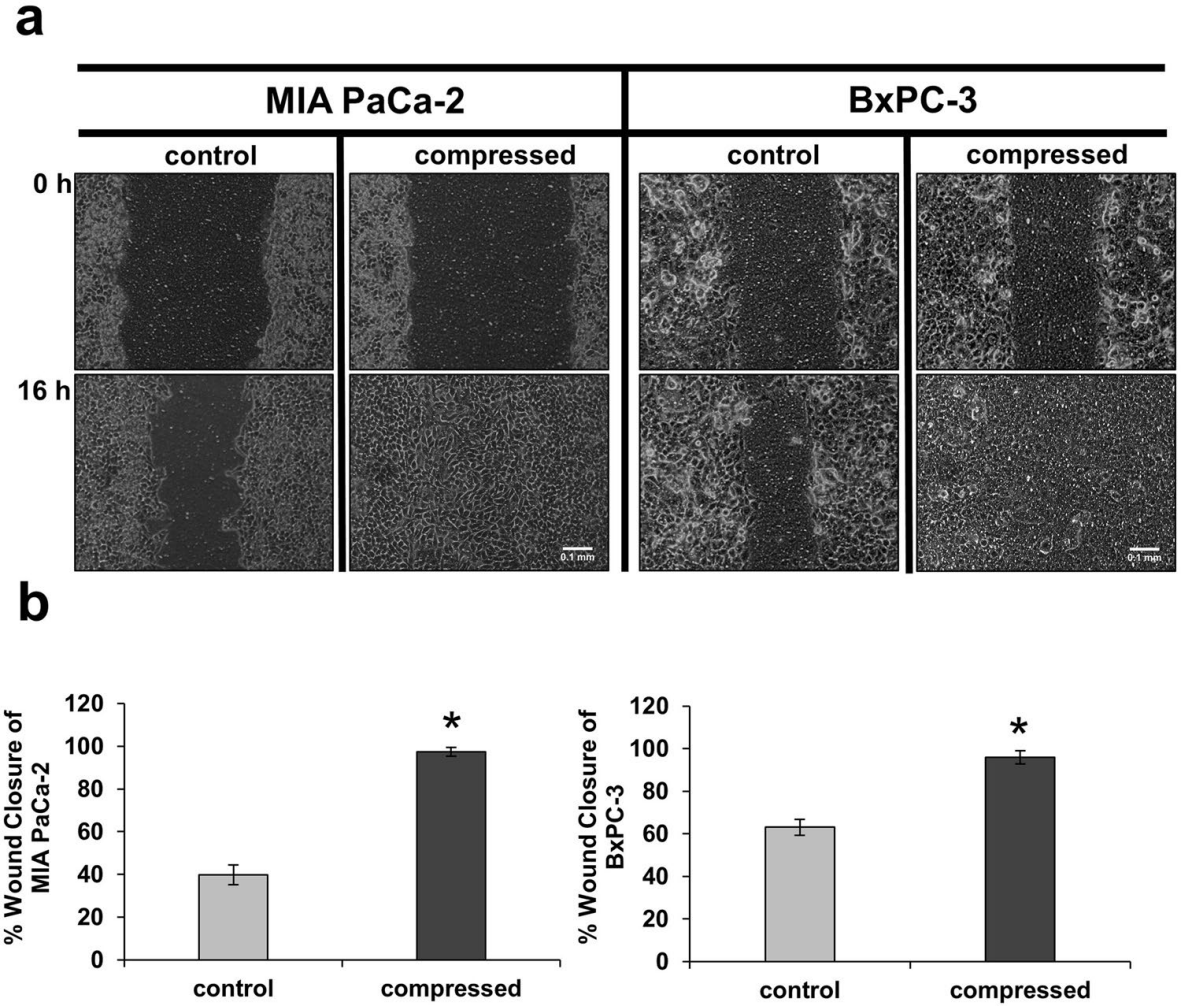
Mechanical Compression promotes pancreatic cancer cell migration. (**a**) MIA PaCa-2 (left) and BxPC-3 (right) pancreatic cancer cells were subjected to a scratch-wound assay under 4.0 mmHg of compressive solid stress for 16 hours. Uncompressed cells (control) were covered with an agarose layer only. Scale bar: 0.1 mm. (**b**) Graphs represent the wound closure of MIA PaCa-2 (left) and BxPC-3 (right) as analyzed by ImageJ software. In each condition, at least four different images from two independent experiments were analyzed. Statistically significant differences (p < 0.05) between compressed and uncompressed MIA PaCa-2 or BxPC-3 are indicated with asterisk (*).

It has been previously reported that mechanical compression promotes breast and colon cancer cell migration and invasion^6,8,9^, whereas there is no information on the effect of it on pancreatic cancer cells. In the present study, we used MIA PaCa-2 and BxPC-3 pancreatic cancer cell lines to study their migratory ability as a response to mechanical compression. Cells were compressed at 4.0 mmHg, which is similar in magnitude to the stress levels measured *in* situ by Nia *et al*., (Fig. 1D in ref. ^21^), and subjected to a scratch wound healing assay. Although this experimental approach presents some technical hurdles involving critical steps such as generation of the wound, placement of the agarose cushion, application of mechanical pressure, and removal of the agarose cushion without damaging the cells, it proved to be ideal for our study as it offered the advantage of allowing us to accurately assess the migratory ability of cancer cells under mechanical compression. As shown in Fig. 1a,b, both cell lines exhibited increased migratory ability as a response to mechanical compression. Notably, cell viability of compressed compared to uncompressed cells was not compromised by the applied pressure, indicating that cells were viable and healthy despite the mechanical load (Supplementary Fig. 1b).

### Mechanical Compression stimulates GDF15 secretion and upregulation of Rho GTPases mRNA expression

**Figure 2:**
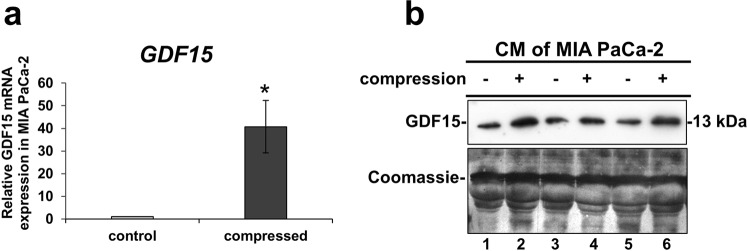
Mechanical Compression stimulates the mRNA expression and secretion of GDF15. (**a**) MIA PaCa-2 cells were subjected to 4.0 mmHg of compressive stress for 16 hours and the expression of GDF15 was measured by qPCR. The mRNA expression in each sample was quantified by the ΔΔCt method using the expression in uncompressed cells as a reference. Bar graphs represent the mean fold change ± SE of four biological replicates (n = 12). Statistically significant changes between compressed and uncompressed cells are indicated by an asterisk (*) (p < 0.05). **(b)** Western Blotting showing the secretion of GDF15 in the conditioned medium (concentrated by 40X) of compressed MIA PaCa-2 from three independent experiments. Coomassie staining was used to verify equal protein loading. Full length blot can be found in Supplementary Fig. 6a.

Intrigued by our previous work, showing that GDF15 is strongly upregulated in normal pancreatic fibroblasts as a response to mechanical compression^12^, and knowing that GDF15 as well as Rho GTPases are upregulated in response to mechanical stretch or cytoskeleton disruption^22–24^, we examined the gene expression of these genes in compressed MIA PaCa-2 and BxPC-3 cells. We observed a strong increase in *GDF15* and *RhoB* mRNA expression (Fig. 2a, Supplementary Figs 2 and 3a) and elevated GDF15 secretion in the conditioned medium (Fig. 2b, Supplementary Fig. 3b) of both cell lines with MIA PaCa-2 cells exhibiting the most dramatic changes.

### GDF15 is a key regulator for solid stress-induced pancreatic cancer cell migration

**Figure 3:**
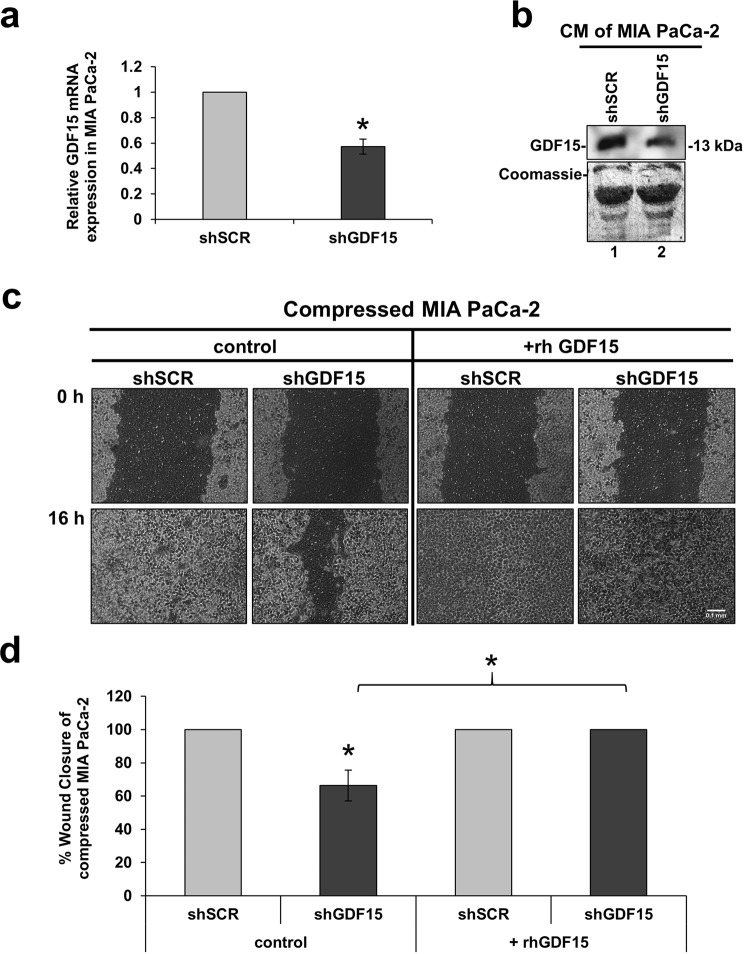
GDF15 is a key regulator for solid stress-induced pancreatic cancer cell migration. (**a**) MIA PaCa-2 cancer cells were transiently transfected with shSCR- and shGDF15- expressing vectors and were compressed by 4.0 mmHg in 2% FBS containing DMEM. Total RNA was then isolated and *GDF15* mRNA expression was quantified by qPCR. Each bar indicates the mean fold change ± SE of a representative experiment (n = 3). Asterisk (*) indicates a statistically significant difference (p < 0.05). **(b)** Representative Western Blotting showing that GDF15 secretion has been succesfully reduced in the conditioned medium (40X concentrated) of compressed shGDF15-treated MIA PaCa-2 cells (lane 2) compared to compressed shSCR cells (lane 1). Full length blot can be found in Supplementary Fig. 6b. **(c)** MIA PaCa-2 cells knockdown for GDF15 were compressed by 4.0 mmHg in low-serum medium and then subjected to a scratch wound healing assay stimulated by 10 ng/ml rhGDF15 for 16 hours. Control cells (shSCR) were treated with solvent (indicated as control). Scale bar: 0.1 mm. **(d)** Graph showing the percentage wound closure as quantified using ImageJ software. Statistical significant difference in wound closure of shGDF15 MIA PaCa-2 cells compared to shSCR MIA PaCa-2 cells both treated with solvent (control) is indicated with an asterisk (*) (n = 4; p < 0.05).

In order to identify how GDF15 is implicated in cancer cell migration under solid stress conditions, it was transiently silenced using an shRNA or siRNA-mediated silcening approach. Mechanical compression was then applied for 16 hours. As shown in Fig. 3 and Supplementary Fig. 4a,b, *GDF15* was effectively depleted both at the mRNA and protein level following each silencing approach (shRNA or siRNA) (Fig. 3a,b, Supplementary Fig. 4a,b). With regard to cell migration, our results show that MIA PaCa-2 cells lacking GDF15 have reduced migratory ability (Fig. 3c,d, Supplementary Fig. 4c,d) indicating that *GDF15* is critically involved in solid stress-induced pancreatic cancer cell migration. Interestingly, however, treatment with rhGDF15 managed to completely reverse this inhibitory effect (Fig. 3c,d), clearly suggesting that *GDF15* plays a crucial role in solid stress-induced pancreatic cancer cell migration.

### Screening for the identification of solid stress signal transduction mechanisms

**Figure 4:**
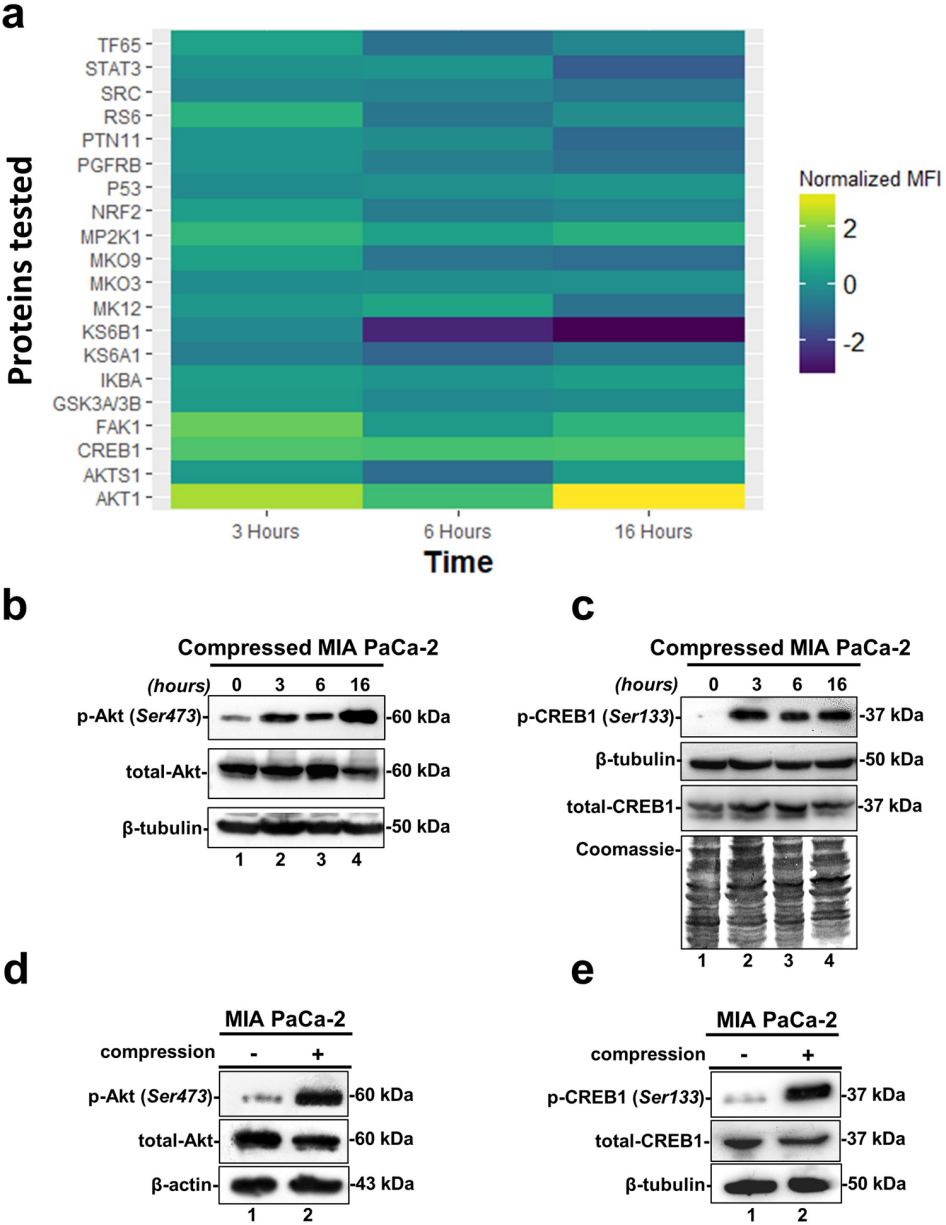
Screening for identification of solid stress signal transduction mechanisms. (**a**) The heatmap depicts the change of the normalized Median Fluorescent Intensity (MFI) for the compressed cells at 3, 6 and 16 hours compared to the MFI for the uncompressed cells**. (b**,**c)** Validation of Akt phosphorylation (Ser473) and CREB1 phosphorylation (Ser133). B-tubulin (B) or a Coomassie staining (C) have been used as loading controls. Full length blots can be found in Supplementary Fig. 6c,d. **(d,e)** Western Blotting showing Akt and CREB1 phosphorylation levels in MIA PaCa-2 compressed by 4.0 mmHg for 16 hours. B-actin (D) or β-tubulin (E) were used as loading controls. Full length blots can be found in Supplementary Fig. 6e,f.

Based on the fact that mechanical compression regulates gene expression of pancreatic cancer cells, we investigated the mechanism by which the extracellular mechanical stimuli are transmitted into the cell nucleus ultimately regulating cellular responses. To that regard, we applied mechanical compression on MIA PaCa-2 cells, that exhibited the most dramatic changes, for different time points, and whole cell lysates were screened for the identification of activated signaling pathways by using Multiplex Assay designed to detect 24 influential phospho-proteins. Analysis of the Multiplex Assay findings showed that Akt was strongly upregulated by mechanical compression as early as 3 hours post compression, as indicated by the increased phosphorylation level of Akt (Ser473 residue) (Fig. 4a). Strikingly, among all phosphoproteins tested in this screen, Cyclic AMP-responsive element-binding protein-1 (CREB1), which is directly linked to Akt phosphorylation as a widely known transcription factor downstream of Akt^25–27^, was also found to be activated in compressed cells (phosphorylation on Ser133 residue) (Fig. 4a). Akt and CREB1 activation was verified by Western Blotting with the maximum increase observed at 16 hours (in Fig. 4b,c compare lane 4 to lane 1), which corresponds to the time point at which cells closed the wound in the wound healing assay (Fig. 1a). To validate the results of our initial screen and time-point experiment, we ran an independent experiment where MIA PaCa-2 and BxPC-3 were compressed for 16 hours and confirmed that Akt/CREB1 pathway gets activated in response to mechanical compression at the specific time point (Fig. 4d,e, for MIA PaCa-2 and Supplementary Fig. 3c, for BxPC-3). Our data suggest that this pathway mediates a solid stress signal transduction mechanism that could regulate cancer cell migration^28,29^.

### Akt pathway is required for solid stress-induced pancreatic cancer cell migration

**Figure 5:**
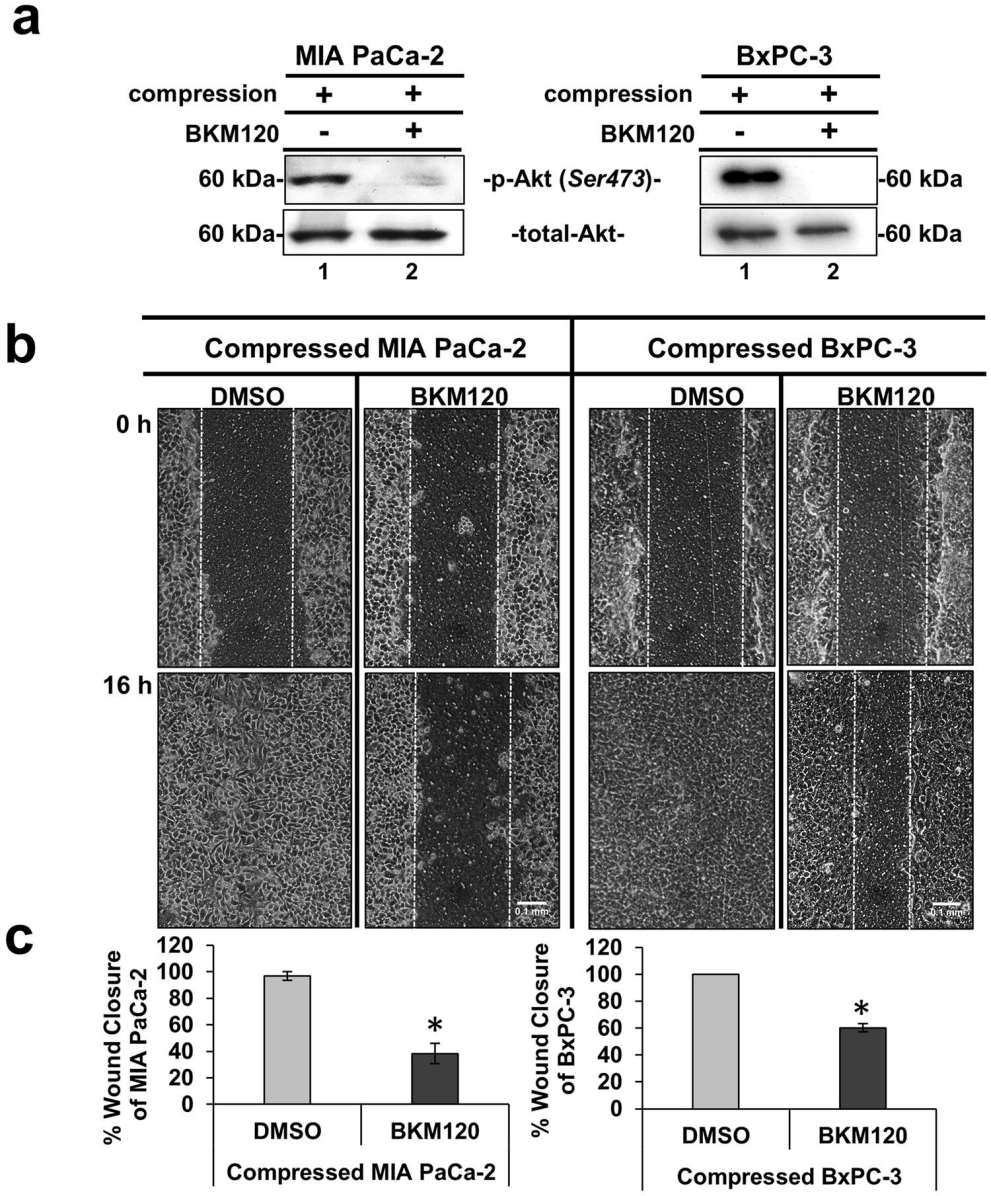
Akt pathway is required for solid stress-induced pancreatic cancer cell migration. (**a**) MIA PaCa-2 and BxPC-3 were pre-treated with 10 μΜ BKM120 for 1 hour and then were compressed by 4.0 mmHg for 16 hours in 2% FBS-containing medium. Control cells were treated with equal volume of DMSO. Proteins were extracted and Western Blotting represents the levels of Akt phosphorylation (Ser473) in MIA PaCa-2 (left) and BxPC-3 (right). Full length blot can be found in Supplementary Fig. 6i. **(b)** MIA PaCa-2 and BxPC-3 were pre-treated with 10 μΜ BKM120 for 1 hour, and then were subjected to a scratch wound assay under 4.0 mmHg in 2% FBS-containing medium for 16 hours. Control cells were treated with equal volume of DMSO. Scale bar: 0.1 mm. White dashed line indicates the difference in wound closure between 0 and 16 hours. **(c)** Graphs represent the wound closure between compressed MIA PaCa-2 (left) or BxPC-3 (right) treated with DMSO compared to compressed cells treated with 10 μΜ BKM120 as quantified using the ImageJ software. Two independent experiments were performed and at least four different images were analyzed. Statistical significant differences are indicated with an asterisk (*) (p < 0.5).

**Figure 6:**
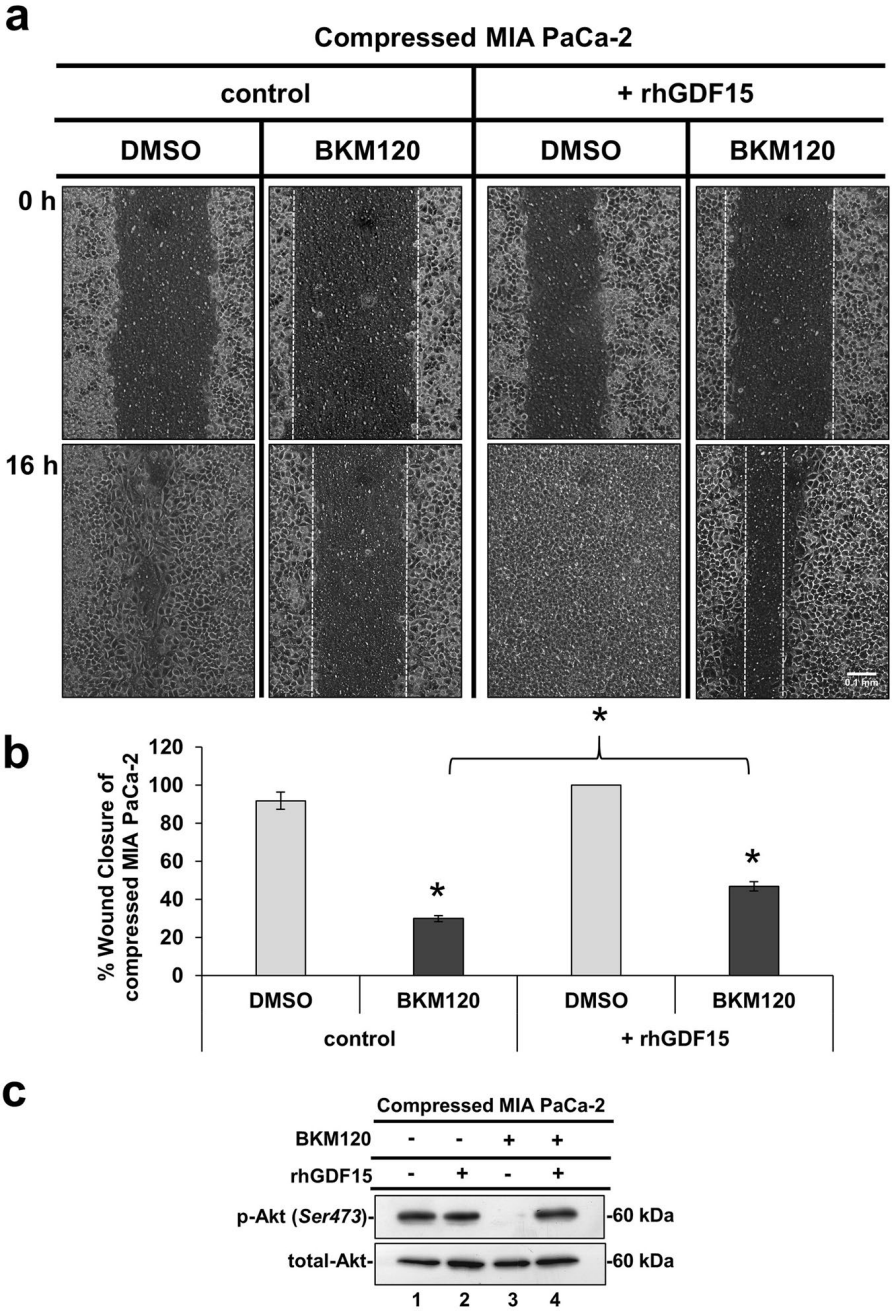
Treatment with rhGDF15 can overcome the blockade of solid stress-induced migration caused by Akt inhibition. (**a**) MIA PaCa-2 were pre-treated with 10 μΜ ΒΚΜ120 or equal quantity of DMSO and subjected to a scratch wound healing assay under 4.0 mmHg of compression in the presence of 10 ng/ml rhGDF15 or equal quantity of the respective solvent (control). Pictures from 4 different fields were taken from two independent experiments. Scale bar: 0.1 mm. White dashed line shows the difference in wound closure between 0 and 16 hours. (**b**) Graph showing the perentage wound closure of compressed MIA PaCa-2 treated with DMSO or 10 μΜ BKM120 in the presence of 10 ng/ml rhGD15 or solvent (control). Asterisk (*) indicates a statistical significant difference between compressed MIA PaCa-2 treated with BKM120 compared to compressed MIA PaCa-2 treated with BKM120 in the presence of rhGDF15 (p < 0.05). **(c)** Representative Western Blotting showing phosphorylated Akt (Ser 473) and total Akt levels in compressed MIA PaCa-2 cells treated with 10 ng/ml rhGDF15 or solvent in combination with 10 μΜ BKM120 or DMSO. Full length blots can be found in Supplementary Fig. 6j.

The Akt pathway is a well characterized signaling pathway known to play a crucial role in cell survival and inhibition of apoptosis^29^. Interestingly however, several studies have also shown that this pathway can be responsible for cancer cell migration and invasion^11,18,25,30–32^. Nevertheless, the involvement of the Akt pathway in solid stress-induced pancreatic cancer cell metastasis has not been described yet. To this end, we used an inhibitor of Phosphoinositide 3-kinase (PI3K), which is directly upstream of Akt to test the solid stress-induced migration with or without activated Akt. BKM120 (NVP-BKM120 or Buparlisib), is a PI3K inhibitor that has been previously used to inhibit Akt activation *in vitro* and *in vivo*^33–37^. Thus, we applied mechanical compression on MIA PaCa-2 and BxPC-3 cells treated with BKM120 and we verified that Akt phosphorylation was successfully inhibited in both cell lines (Fig. 5a), without any significant effect on cell viability (Supplementary Fig. 1c). Moreover, we observed that the migratory ability of both cell lines was also blocked (Fig. 5b,c) suggesting a critical involvement of Akt in this process. Finally, we wanted to test whether rhGDF15, which is shown to promote pancreatic cancer cell migration (Fig. 3), could rescue the Akt inhibitory effect. We observed that while BKM120 blocked MIA PaCa-2 migration, treatment with rhGDF15 partially reversed this phenotype (Fig. 6a,b). The same effect was observed in the activation of Akt pathway, since rhGDF15 was able to rescue Akt phosphorylation levels blocked by BKM120 (Fig. 6c). These results, suggest that GDF15 could activate Akt pathway and act synergistically to promote pancreatic cancer cell migration under mechanical compression (Fig. 6a–c).

### Solid stress signal transduction is mediated by Akt/CREB1 pathway to regulate GDF15 expression

**Figure 7:**
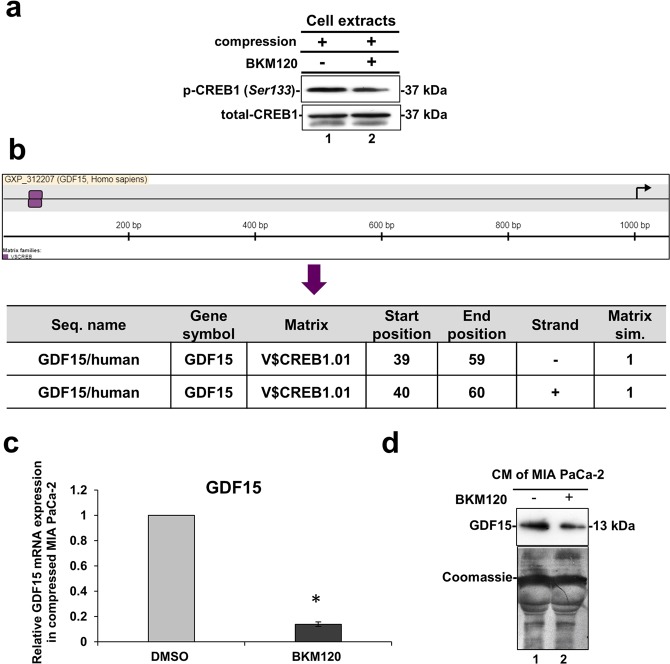
Solid stress signal transduction is mediated by Akt/CREB1 to regulate GDF15 expression. (**a**) Representative Western Blotting showing phosphorylated CREB1 (Ser 133) and total CREB1 levels in compressed MIA PaCa-2 cells treated with 10 μΜ BKM120 or DMSO. Full length blots can be found in Supplementary Fig. 6k. (**b**) Prediction of CREB1 (Matrix) trascription factor-binding sites on the nucleotide sequence of GDF15 (Seq. name) as predicted by MatInspector tool. (**c**) qPCR was used to quantify the mRNA levels of *GDF15* in compressed MIA PaCa-2 cells treated with 10 μΜ BKM120 compared to compressed cells treated with DMSO. ΔΔCt method was used to quantify the gene expression in each sample using as a reference the expression in compressed and treated with DMSO cells (control). Bar graphs represent the mean fold change ± SE of two independent experiments (n = 6) and statistical changes are indicated with an asterisk (*) (p < 0.05). (**d**) Western Blotting showing that GDF15 secretion in the conditioned medium (concentrated by 40X) of compressed MIA PaCa-2 cells treated with BKM120 (lane 2) is reduced compared to compressed cells treated with DMSO (lane 1). Coomassie staining was used to ensure equal protein loading. Full length blot can be found in Supplementary Fig. 6l.

We next wondered whether Akt regulates the expression of factors responsible for pancreatic cancer cell migration through CREB1 phosphorylation. As presented in Fig. 7a, CREB1 phosphorylation was also inhibited in compressed MIA PaCa-2 in the presence of BKM120, suggesting that Akt activation is necessary for the subsequent CREB1 activation. Hence, to determine whether CREB1 regulates the expression of factors responsible for pancreatic cancer cell migration, we searched for CREB1-binding sequences using the MatInspector tool (Genomatix)^38^, which identifies transcription-factor-binding sites in nucleotide sequences using a large library of weight matrices (Matrix Family Library version 11.0). We found that CREB1 can bind at two sites on the 5′ promoter region of *GDF15* exhibiting a perfect match getting a matrix similarity of 1.00 (which is translated to 100% match between Transcription factor-binding site and nucleotide sequences) (Fig. 7b). Thus, we analyzed the mRNA expression of *GDF15* in compressed MIA PaCa-2 cells treated with BKM120. Interestingly, qPCR analysis revealed that *GDF15* mRNA expression as well as GDF15 secretion were downregulated when Akt/CREB1 pathway was blocked by BKM120 (Fig. 7c,d). This result suggests that the Akt/CREB1 pathway is activated by solid stress in order to regulate the expression of *GDF15* mediating pancreatic cancer cell migration.

## Discussion

It has been well established that biomechanical cues have a dominant role in regulating tumor aggressiveness^1,2,39^. For instance, fluid shear stress, acting on a cell surface due to the vascular and interstitial fluid flow within and around a tumor tissue, is a typical biomechanical factor that has been suggested to promote invasion and metastasis of cancer cells to distant organs^40–42^. In a similar manner, solid stress, generated by the solid components of a tumor, has been found to affect cancer cell migration and promote tumor progression^1,2^. However, further studies are required to understand how physical factors are translated into biomechanical signals and finally regulate cellular behaviour.

In the current study, we used an *in vitro* compression device to apply predefined and measurable compressive solid stress, similar in magnitude to the stress applied on cells in the tumor interior. The level of compressive stress used in this study (4.0 mmHg) was in agreement with experimental estimations of solid stress in pancreatic tumor models^21^, and it is used herein to enhance the metastatic potential of MIA PaCa-2 and BxPC-3 pancreatic cancer cell lines, as indicated by a scratch wound assay (Fig. 1a,b), and to upregulate *GDF15* expression and secretion, as indicated by qPCR and Western Blotting (Fig. 2 and Supplementary Fig. 3). Furthermore, we show that compressed MIA PaCa-2 cells lacking *GDF15* (either after shGDF15 or siGDF15 treatment) exhibited reduced migratory ability while treatment with rhGDF15 reversed this effect, suggesting that GDF15 plays a crucial role in solid stress-induced pancreatic cancer cell migration (Fig. 3 and Supplementary Fig. 4). These novel findings could possibly give an explanation for the elevated GDF15 levels observed in the serum of patients with solid tumors, such as in the case of breast, prostate and pancreatic cancer that exhibit high solid stress levels^21,39^.

In order to identify possible solid stress signal transduction mechanisms that regulate gene expression in cancer cells, we screened compressed MIA PaCa-2 cells, which showed the most robust mRNA changes, for activated signaling pathways implicated in mechanical stimuli signal transduction. We found that, from the pathways tested, the Akt pathway was activated as a response to solid stress as early as 3 hours post-compression, suggesting that solid stress could by itself activate this pathway. Furthermore, we demonstrated that inhibition of Akt pathway by the PI3K inhibitor, BKM120, completely blocked the compression-induced migration of cancer cells (Fig. 5). It should be noted that PI3K/Akt has been found upregulated in 59% of patients with pancreatic cancer, while BKM120 has been already used in studies employing mouse models or is currently being administered in phase I clinical trials for the treatment of patients with solid tumors, such as pancreatic, colon and breast tumors^34–37^, where GDF15 serum levels are found elevated^16^.

Based on the phosphoproteins screen (Fig. 4a), the compression-induced Akt activation could be directly linked to CREB1 activation, as it is an already known downstream transcription factor of Akt pathway^25–27^. This hypothesis was verified by the inhibition of CREB1 phosphorylation in compressed MIA PaCa-2 cells treated with BKM120 (Fig. 7a). Interestingly, by using the MatInspector tool, we also found that CREB1 has two binding sites on *GDF15* promoter (Fig. 7b). To this end, we showed that GDF15 mRNA expression and secretion is downregulated in compressed MIA PaCa-2 cells treated with BKM120 (Fig. 7c,d), suggesting a novel regulatory mechanism of GDF15 by Akt/CREB1 pathway under compression. In order to examine whether there is a positive feedback loop between GDF15 and Akt/CREB1 pathway, we treated uncompressed MIA PaCa-2 cells with rhGDF15 and found that Akt/CREB1 pathway (Supplementary Fig. 5a) is activated, while in the absence of GDF15 Akt is blocked without any change in CREB1 phosphorylation levels as previously shown^18,43,44^ (Supplementary Fig. 5b). Notably, compressed MIA PaCa-2 lacking GDF15 showed increased phosphorylation levels of Akt, but not of CREB1 (Supplementary Fig. 5b), suggesting that other pathways are activated in the absence of GDF15, in order to regulate Akt pathway either in compressed or uncompressed conditions.

**Figure 8:**
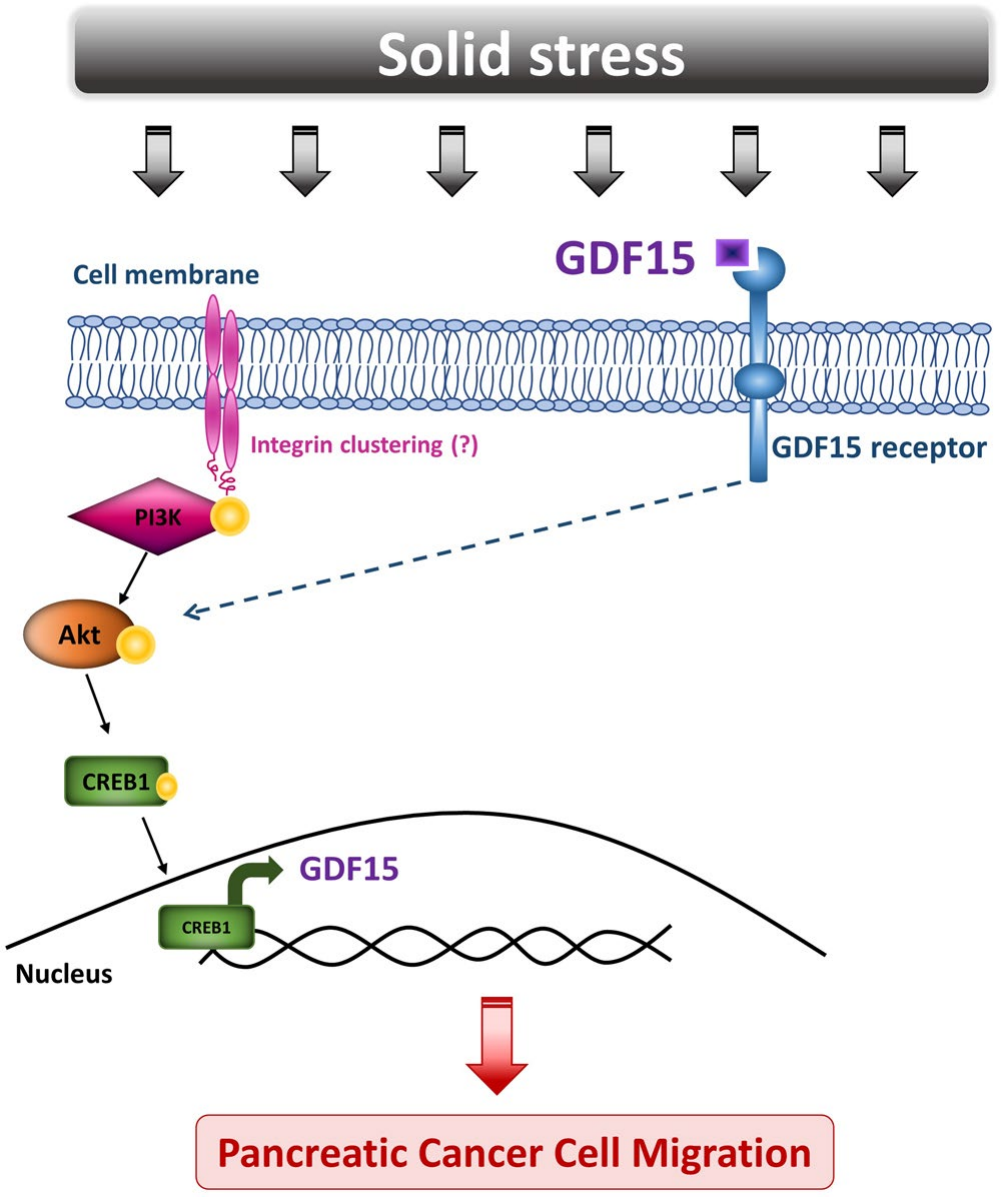
Proposed mechanism of how solid stress signal transduction via Akt pathway regulates GDF15 expression to induce pancreatic cancer cell migration. The development of solid stress during the growth of several solid tumors, such as pancreatic cancer, activates Akt pathway which in turn phosphorylates CREB1. Subsequently, the activated CREB1 acts as transcription factor by a direct binding onto the promoter region of GDF15. GDF15 is then secreted and acts in an autocrine manner to promote pancreatic cancer cell migration.

In conclusion, we propose a model suggesting that solid stress signal transduction is mediated by Akt/CREB1 pathway to transcriptionally regulate *GDF15* expression. Subsequently, GDF15 is secreted and acts in an autocrine manner to promote pancreatic cancer cell migration possibly through Akt activation (Fig. 8). Although many questions still remain regarding the exact molecular mechanism involved in solid stress-induced migration, this is the first study actually connecting solid stress-induced migratory profile of cells with GDF15 upregulation and secretion through Akt/CREB1 activation, bringing GDF15 to the centre of solid tumor biology and rendering it a potential target for future anti-metastatic therapeutic innovations.

## Materials and Methods

### Cell culture

Pancreatic cancer cell lines, MIA PaCa-2 and BxPC-3, were obtained from American Type Culture Collection (ATCC) and were incubated at 37 °C and 5% CO_2_ in a humidified incubator. MIA PaCa-2 cells were maintained in Dulbecco’s Modified Eagle’s Medium (DMEM) and BxPC-3 cells in Roswell Park Memorial Institute 1640 (RPMI) medium, both supplemented with 10% Fetal Bovine Serum (FBS) and 1% antibiotics.

### *In vitro* compression device

In order to apply a defined and controlled compressive solid stress on cancer cells, we used a previously described experimental setup, known as transmembrane pressure device^1,5,8,11,12,20^. In short, 2 × 10^5^ cells were seeded in the upper chamber of a 24 mm diameter transwell insert with 0.4 μm pores (Greiner Bio-one) and a layer of 2% agarose gel was placed on top of the cells as shown in Supplementary Fig. 1a. A piston of a desired weight was placed on the top of the agarose cushion and the cells were compressed by 4.0 mmHg for 16 hours or to 4.0 mmHg stress for 3, 6 and 16 hours. Uncompressed cells (control) were covered with an agarose cushion only (i.e., 0.0 mmHg). The solid stress level used in this study was selected according to the levels estimated in pancreatic tumors^21^.

### Transient transfection of pancreatic cancer cells with shRNA against *GDF15*

To deplete *GDF15* from pancreatic cancer cells, we generated vectors that express shRNA against *GDF15* (shGDF15) and control vectors (sh-Scrambled or shSCR) as described in detail in the Supplementary Material. Cells were transiently transfected with each shRNA using Lipofectamine 2000 transfection reagent (Invitrogen) according to manufacturer’s guidelines.

### *In vitro* Scratch assay

Cancer cells were grown to form a monolayer and a cross-like wound was created using a 200 μL pipette tip. Cells were then washed with Phosphate Buffered Saline (PBS) and treated as indicated in each figure caption for 16 hours. Pictures from four different fields per condition were captured at 0 and 16 hours. The wound area was calculated by the ImageJ software and quantification was performed using the following equation:

$$(({\rm{Wound}}\,{\rm{area}}\,{\rm{at}}\,{\rm{0}}\,{\rm{hours}}-{\rm{Wound}}\,{\rm{area}}\,{\rm{at}}\,16\,\mathrm{hours})/({\rm{Wound}}\,{\rm{area}}\,{\rm{at}}\,0\,\mathrm{hours}))\times 100.$$

### Cell treatments

In order to examine the effect of GDF15 on the migratory ability of pancreatic cancer cells under 4.0 mmHg solid stress condition, MIA PaCa-2 cells lacking *GDF15 (*shGDF15) or control cells (shSCR) were grown in transwell inserts and were subjected to a scratch wound healing assay in the presence or absence of 10 ng/ml of human recombinant GDF15 (rhGDF15, R&D systems) under mechanical compression in 2% FBS-containing medium for 16 hours. Control cells were treated with equal volume of the solvent used to dissolve rhGDF15 (4 mM HCl supplemented with 0.1% Bovine Serum Albumin-BSA). In order to study the role of Akt pathway in pancreatic cancer cell migration under mechanical compression, a PI3K inhibitor (BKM120 or Buparlisib, MedChemExpress) was selected to block the Akt pathway. Cells were grown in transwell inserts with 2% FBS-containing medium for 24 hours and were then pre-treated with 10 μΜ BKM120 or equal volume of DMSO for 1 hour. The concentration of BKM120 was selected according to the manufacturer’s guidilines. Mechanical compression (4.0 mmHg) was then applied and a wound healing assay was performed. To study the role of Akt pathway and GDF15 on the migration of pancreatic cancer cells, MIA PaCa-2 cells were pre-treated with 10 μΜ BKM120 or equal volume of DMSO for 1 hour and subjected to a scratch wound healing assay under mechanical compression (4.0 mmHg) in the presence of 10 ng/ml rhGDF15 or equal volume of solvent.

### Cell Viability Assay

Cell viability of cancer cells was assesed using Alamar Blue reagent (Invitrogen Life Technologies) following compression with or without treatment with BKM120 inhibitor, following the manufacturer’s instructions.

### Gene expression analysis

RNA isolation from cancer cells was performed using Trizol (Invitrogen). RNA was then converted into cDNA using Superscript Reverse Transcriptase (Invitrogen). Gene expression was quantified by real-time PCR using SYBR Green Supermix (KAPA Biosystems) in a real-time PCR machine (BioRad). The expression of each gene was measured in triplicate and normalized with the *β-actin* housekeeping gene. The ΔΔCt method was then used to quantify the gene expression of each condition with a respective calibrator as specified in each figure legend. The primers used for each target gene are shown in Supplementary Table 1.

### Phosphoproteomics

24 custom dual-antibody Luminex assays were developed using ProtATonce (Athens, Greece) multiplex assay service. Briefly, 2 to 5 antibodies were selected and tested pair-wise as capture and as detection antibody. For each protein, the optimal capture/detection antibody pair was selected based on signal-to-noise ratio measurement. Assays were then multiplexed and concentration of detection antibody was evaluated based on its signal and its noise (off-target signal) in the panel.

### Sample preparation and phosphoprotein’s measurements

Cells were plated in transwell inserts and were compressed with 4.0 mmHg stress for 3, 6 and 16 hours in 2% FBS-containing DMEM. Protein isolation was performed using radio immunoprecipitation assay (RIPA) buffer containing a protease inhibitor cocktail tablet (Sigma). The BCA protein assay kit (Pierce) was used to determine protein concetration and 200 μg/ml of cell lysates were used for phosphoprotein measurements. 24 capture antibodies coupled to Luminex magnetic beads and 24 biotinylated detection antibodies were multiplexed to create the bead mix and the detection mix, respectively. 50 ul of the coupled beads (bead mix) were incubated with the samples on a flat bottom 96-well plate on a shaker at 900 rpm for 90 minutes at room temperature. Then, detection mix was added, and the samples incubated on a shaker at 900 rpm for 60 minutes at room temperature. The final step was the addition of freshly prepared SAPE solution (Streptavidin, R-Phycoerythrin conjugate, Cat Nr: S866, Invitrogen) for the detection of the signal. 15 minutes after the incubation with SAPE, samples were measured with the Luminex FlexMAP 3D instrument. The exact phospho-proteins tested and the normalization procedure can be found in detail in Supplementary Material.

### Immunoblotting

Whole protein cell lysates were extracted using RIPA buffer containing protease and phosphatase inhibitors. Conditioned medium (~4 ml) was collected after each experiment, filtered through 0.2 μm pores, concentrated 40X using 10 K protein concentrators (Pierce) and stored at −80 °C until use. Protein concentration was determined using the BCA protein assay kit (Pierce) and about 20–40 μg of protein were separated on a 12% SDS-PAGE. Proteins were transferred to a PVDF membrane which was then blocked in 5% non-fat milk or BSA in TBS-T buffer and incubated with anti-Growth Differentiation Factor 15 (GDF15) (Cell Signalling), anti-phospho-Akt (Ser 473) (Cell signalling), anti-Akt total (Cell Signalling), anti-phospho-CREB1 (Ser 133) (Abcam) or anti-CREB1 total (Abcam) antibodies overnight. A Coomassie staining (Sigma), for blots with conditioned medium, or antibodies against β-tubulin or β-actin, for blots with cell extracts, were used as loading controls. The detection of antibodies was performed with enhanced chemiluminescent system (Pierce) using Kodak Biomax light films. Films were then scanned using an HP Scanjet G4010 scanner and images were analyzed in Adobe Photoshop. Color was discarded and images were converted to grayscale. In some cases contrast was used in the entire blot to enhance image clarity. No other image manipulation was performed. Uncropped images of Western Blotting used in this article can be found in Supplementary Fig. 6 as indicated in each figure legend.

### Statistics

Data are expressed as mean ± standard error (SE). Statistical significance was examined by Student’s *t*-test using two-tail distribution. Tests with *p*-values < 0.05 were considered as significantly different and are nominated in each figure with an asterisk (*).

## Supplementary information

Supplementary Material

## Data Availability

All data generated or analyzed during this study are included in this published article and its supplementary information files are available from the corresponding author on reasonable request.
