# Supplementary material for: Solid stress-induced migration is mediated by GDF15 through Akt pathway activation in pancreatic cancer cells
Source: Sci Rep. 2019 Jan 30;9:978. doi: 10.1038/s41598-018-37425-6 (PMC6353927; doi:10.1038/s41598-018-37425-6)
Supplement: Supplementary file 1 — Supplementary Material [file 41598_2018_37425_MOESM1_ESM.pdf]

Solid stress-induced migration is mediated by GDF15 through Akt pathway activation in pancreatic cancer cells.

Maria Kalli [a], Angeliki Minia[b], Vaia Pliaka[b], Christos Fotis[c], Leonidas G Alexopoulos[b,c], Triantafyllos Stylianopoulos [a, *]

[a] Cancer Biophysics Laboratory, Department of Mechanical and Manufacturing Engineering, University of Cyprus, Nicosia, Cyprus

[b] ProtATonce Ltd, Athens, Greece

[c] Department of Mechanical Engineering, National Technical University of Athens, Athens, Greece

Supplementary Materials and Methods

Cloning of shRNA-expressing vector and transient transfection of pancreatic cancer cells. To generate vectors expressing shRNA against *GDF15*, AgeI/EcoRI-digested pLKO.1-puro vector was ligated with 58-base pair annealed oligos. The sequence of the forward oligo was CCGGGCAAGAACTCAGGACGGTGAACTCGAGTTCACCGTCCTGAGTTCTTGCTTTTTG and sequence for the reverse oligo was AATTCAAAAAGCAAGAACTCAGGACGGTGAACTCGAGTTCACCGTCCTGAGTTCTTGC. Ligated plasmids were transformed into XL10 gold competent bacteria and selected on ampicillin-containing LB agar plates (100 μg/ml). Single colonies were grown for 16 hours in LB broth and plasmids were isolated using a NucleoSpin® Plasmid QuickPure kit (Macherey-Nagel). The presence of each insert was tested by PCR (KAPA Biosystems) using pLKO.1 Forward primer: GGAATAGAAGAAGAAGGTGGA and GDF15 Reverse primer: GCAAGAACTCAGGACGGTGAA. Following verification by DNA sequencing (Macrogen, Netherlands), transient transfection of cancer cells was performed with pLKO-shScrambled vector (or shSCR, used as a control) or pLKO-shGDF15 vector using Lipofectamine 2000 transfection reagent (Invitrogen) according to manufacturer's guidelines. Cells were allowed to grow overnight in antibiotics-free medium before the application of mechanical compression or treatment with human recombinant GDF15 (rhGDF15).

siRNA Transfection. MIA PaCa-2 cells were transfected with 100 nM non-specific control siRNA (siCTRL) or siRNA against GDF15 (Santa Cruz Biotechnology) using Lipofectamine 2000 reagent (Invitrogen Life Technologies). Cells were then compressed by 4 mmHg and a scratch wound assay was performed. Cells were harvested 16 hours post-transfection and silencing efficiency was verified by Western Blotting and Real Time PCR.

GDF15 treatments. In order to examine the effect of GDF15 on Akt pathway activation in pancreatic cancer cells, MIA PaCa-2 cells were grown in 2 % FBS-containing medium for 16 hours. Cells were then stimulated with 10 ng/ml of human recombinant GDF15 (rhGDF15, R&D systems)

for 30 minutes, 1, 2, 3 and 6 hours. Control cells (mock) were treated with equal volume of the solvent used to dissolve rhGDF15 (4 mM HCl supplemented with 0.1 % Bovine Serum Albumin-BSA).

Phosphoproteomics. The following phospho-proteins were measured: mitogen-activated protein kinase 3 (ERK1, Cat Nr: P-MK03-A01), Transcription factor p65 (NF-κB, Cat Nr: P-NFKB-A01), Dual specificity mitogen-activated protein kinase 1 (MEK1, Cat Nr: P-MP2K1-A01), Mitogen-activated protein kinase 12 (p38, Cat Nr: P-MK12-A01), RAC-alpha serine/threonine-protein kinase (AKT1, Cat Nr: P-AKT1-01), Tyrosine-protein phosphatase non-receptor type 11 (PTN11, Cat Nr: P-PTN11-A01), Signal transducer and activator of transcription 3 (STAT3, Cat Nr: P-STAT3-A01), Heat shock protein beta-1 (HSP27, Cat Nr: P-HSPB1-A01), Signal transducer and activator of transcription 5A (STAT5, Cat Nr: -P-STAT5-A01), Cellular tumor antigen p53 (p53, Cat Nr: P-P53-A01), Glycogen synthase kinase-3 alpha/beta (GSK3A/B, Cat Nr: P-GSK3A/B-A01), Mitogen-activated protein kinase 9 (JNK, Cat Nr: P-MK09-A01), 40S ribosomal protein S6 (RS6, Cat Nr: P-RS6-A01), Ribosomal protein S6 kinase beta-1 (p70S6K, Cat Nr: p-KS6B1-A01), Platelet-derived growth factor receptor beta (PDGFRb, Cat Nr: P-PDGFRb-A01), Tyrosine-protein kinase Lck (LCK, Cat Nr: P-LCK-A01), Ribosomal protein S6 kinase alpha-1 (RSK1, Cat Nr: P-KS6A1-A01), Nuclear factor erythroid 2-related factor 2 (NRF2, Cat Nr: P-NRF2-A01), Cyclic AMP-responsive element-binding protein 1 (CREB1, Cat Nr: P-CREB1-A01), Signal transducer and activator of transcription 6 (STAT6, Cat Nr: P-STAT6-A01), Focal adhesion kinase 1 (FAK1, Cat Nr: P-FAK1-A01), Proto-oncogene tyrosine-protein kinase SRC (SRC, Cat Nr: P-SRC-A01), NF-kappa-B inhibitor alpha (Ik-Ba, Cat Nr: P-NFkB-A01), Proline-rich AKT1 substrate 1 (AKTS1, Cat Nr: P-AKTS1-A01).

Normalization of phosphoproteomics measurements.

Limit of detection filtering

For each of the 24 phosphoproteomic measurements a limit of detection was calculated according to the following formula,

$$lod_i = \mu_{blank,i} + 1.65\sigma_{blank,i} \quad (1)$$

Where, lod_i is the limit of detection for each protein i, while $\mu_{blank,i}$ and $\sigma_{blank,i}$ are the mean and standard deviation of the triplicate blanks, for each protein, respectively.

Using the values from equation *(1)* only the phosphoproteins that had a measurement higher that the limit of detection was kept. This filtering approach resulted in the exclusion of HSPB1, STAT5, LCK and STAT6 from further visualization and analysis.

Phosphoprotein MFI normalization

The MFI values for each protein were normalized according to the following formula,

$$MFI_{normalized\ i,j} = 2\ \frac{MFI_{treated\ i,j} - MFI_{control\ i}}{\frac{MFI_{treated\ i,j} + MFI_{control\ i}}{2}} \quad (2)$$

Where, $MFI_{treated\ i,j}$ is the mean fluorescent intensity of the *i-th* phosphoprotein at the *j-th* time point for the compressed cells and $MFI_{control\ i}$ is the mean fluorescent intensity for the *i-th* phosphoprotein for the uncompressed cells.

This normalization procedure was chosen instead of the most commonly used Fold change in order to better visualize the change in MFI for some proteins with low basal MFI (e.g AKT1) (Figure 4).

<u>**Supplementary Figures and Tables**</u>

Supplementary Figure 1. The experimental setup and the cell viability test of compressed MIA PaCa-2. (a) A schematic of the *in vitro* transmembrane pressure device. Cancer cells were grown as a monolayer on the transmembrane of a 0.4 μm transwell insert and a piston of adjustable weight applied a compressive stress. Control cells were covered with an agarose cushion only**. (b)** MIA PaCa-2 were subjected to 4.0 mmHg of compressive stress for 16 hours, with the 0.0 mmHg sample be the negative control. Percentage of cell viability was quantified using the absorbance measured from Alamar Blue assay. No statistically significant differences were observed as compared with the negative control (n=3, *p<0.05). **(c)** MIA PaCa-2 were pre-treated with 10 μM BKM120 or equal volume of DMSO and then subjected to 4.0 mmHg of compressive stress for 16 hours, with the 0.0 mmHg sample being the negative control. Percentage of cell viability was quantified using the absorbance measured from Alamar Blue assay. No statistically significant differences were observed as compared with the negative control (n=3, *p<0.05).

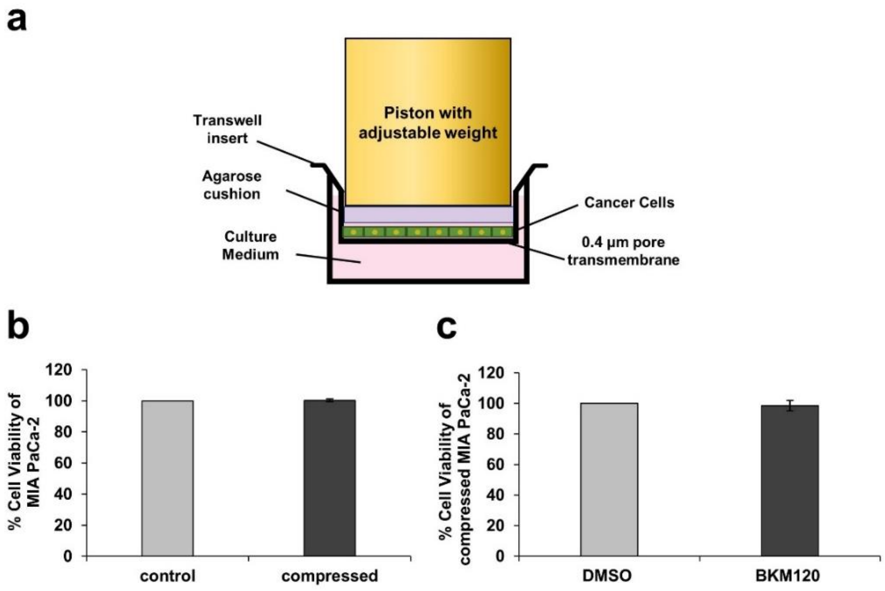

Supplementary Figure 2. MIA PaCa-2 were compressed by 4.0 mmHg for 16 hours and qPCR was used to measure the expression *RhoA* **(a)**, *RhoB* **(b)** and *RhoC* **(c)**. The expression in each sample was analyzed with the ΔΔCt method relative to the expression of control sample (cells compressed by the agarose cushion only). The mean fold change was calculated and plotted for each gene. Each bar indicates the mean fold change ±SE of two independent experiments (n=6). Asterisk (*) indicates a statistically significant difference (p<0.05).

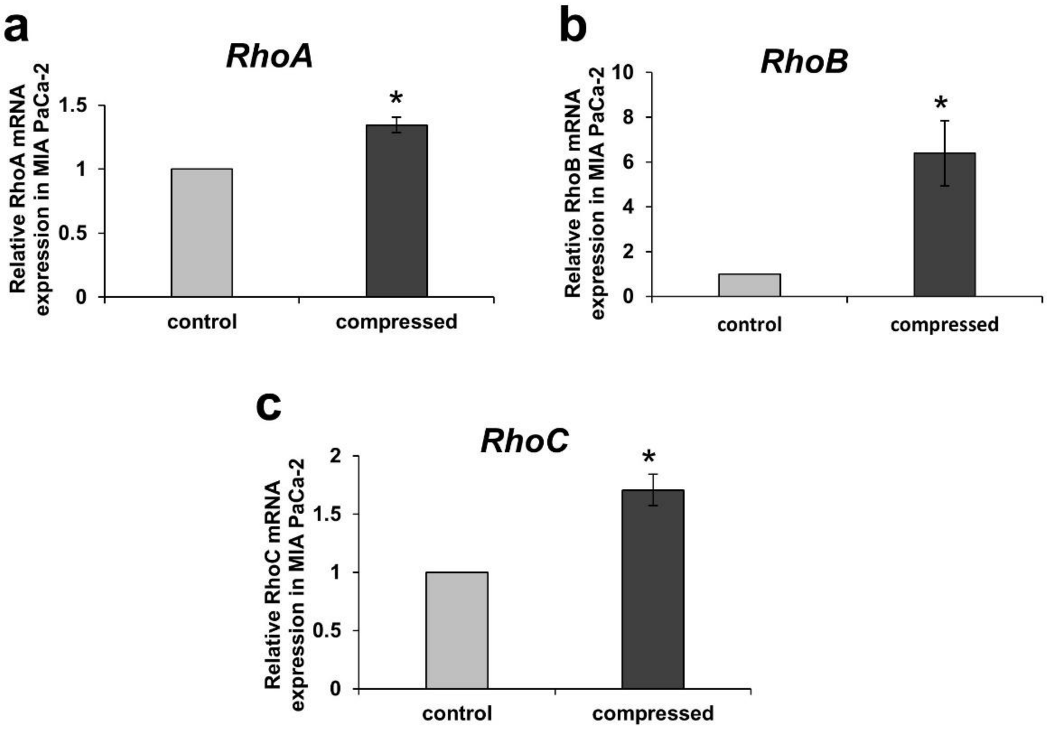

Supplementary Figure 3. (a) BxPC-3 were compressed by 4.0 mmHg for 16 hours and qPCR was used to measure the expression of *GDF15, RhoA, RhoB* and *RhoC.* The expression in each sample was analyzed with the ΔΔCt method relative to the expression of control sample (cells compressed by the agarose cushion only). The mean fold change was calculated and plotted for each gene. Each bar indicates the mean fold change ±SE of two independent experiments (n=6). Asterisk (*) indicates a statistically significant difference ($p<0.05$). **(b)** Western blot showing GDF15 protein levels in the conditioned medium (concentrated by 40 X) of compressed BxPC-3. Coomassie staining was used to verify equal protein loading. **(c)** Western Blotting showing phosphorylated Akt (Ser 473), total Akt, phosphorylated CREB1 (Ser 133) and total CREB1 levels in compressed BxPC-3.

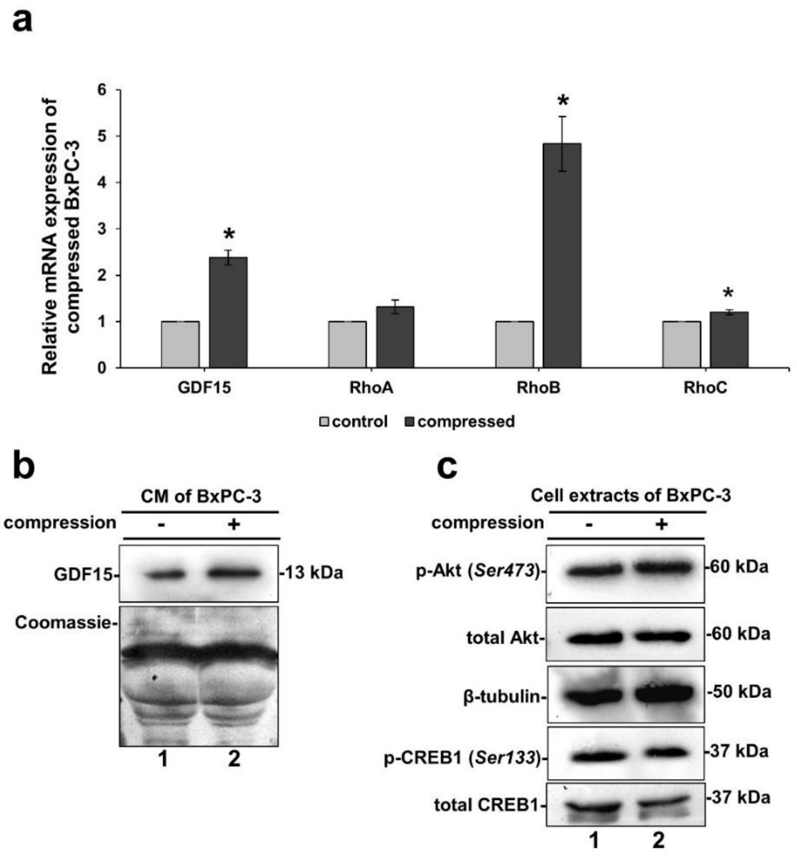

Supplementary Figure 4. Knockdown of GDF15 using siRNA impaired pancreatic cancer cell migration. (a) MIA PaCa-2 cancer cells were transfected with control siRNA (siCTRL) or siRNA against GDF15 (siGDF15) and were compressed by 4.0 mmHg in 2 % FBS containing DMEM. Total RNA was then isolated and *GDF15* mRNA expression was quantified by qPCR. Each bar indicates the mean fold change ±SE of a representative experiment (n=3). Asterisk (*) indicates a statistically significant difference (p<0.05). **(b)** Representative Western blotting showing that GDF15 secretion has been successfully reduced in the conditioned medium (40X concentrated) of compressed siGDF15-treated MIA PaCa-2 cells (lane 2) compared to compressed siCTRL cells (lane 1). Full length blot can be found in **Supplementary Fig. 6m**. **(c)** MIA PaCa-2 cells knockdown for GDF15 were compressed by 4.0 mmHg in low-serum medium and then subjected to a scratch wound healing assay for 16 hours. Scale bar: 0.1 mm. **(d)** Graph showing the percentage of wound closure as quantified using ImageJ software. Statistical significant difference in wound closure of siGDF15-treated MIA PaCa-2 cells compared to siCTRL-treated MIA PaCa-2 cells is indicated with an asterisk (*) (n=4; p<0.05).

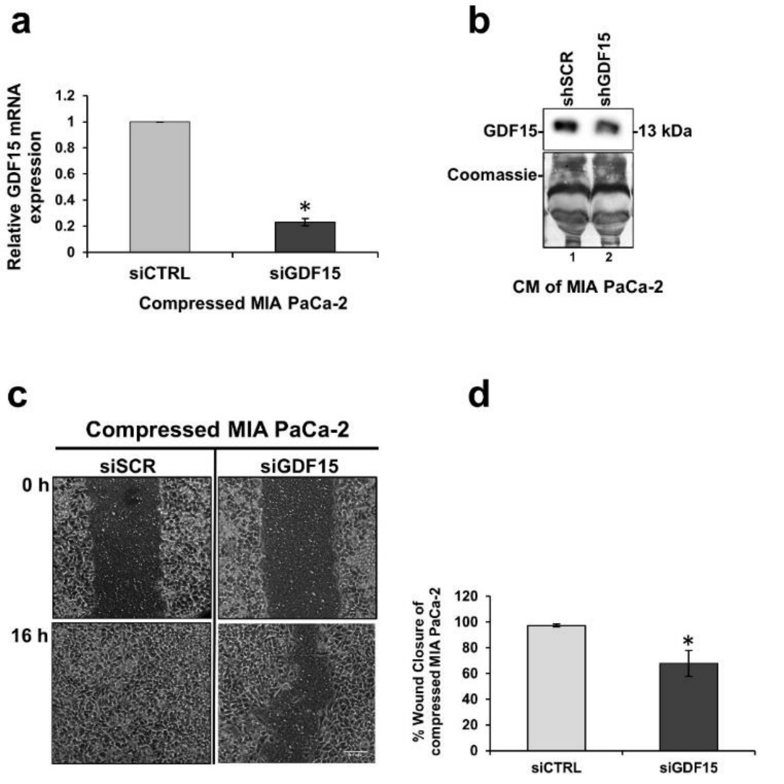

Supplementary Figure 5. Akt pathway is regulated by GDF15 in compressed and uncompressed pancreatic cancer cells. **(a)** MIA PaCa-2 cells were treated with 10 ng/ml human recombinant GDF15 (rhGDF15) for 30 minutes, 1, 2, 3 and 6 hours. Western Blotting was used to determine phosphorylated Akt (Ser 473), total Akt, phosphorylated CREB1 (Ser 133) and total CREB1 levels. Control cells (mock) were treated with equal volume of solvent. Full length blot can be found in Supplementary Fig.6n. **(b)** Western Blotting was used to determine phosphorylated Akt (Ser 473), total Akt, phosphorylated CREB1 (Ser 133) and total CREB1 levels in compressed (right) and uncompressed (left) MIA PaCa-2 cells transiently transfected with shRNA vector against *GDF15*. Full length blots can be found **in Supplementary Fig. 6n**.

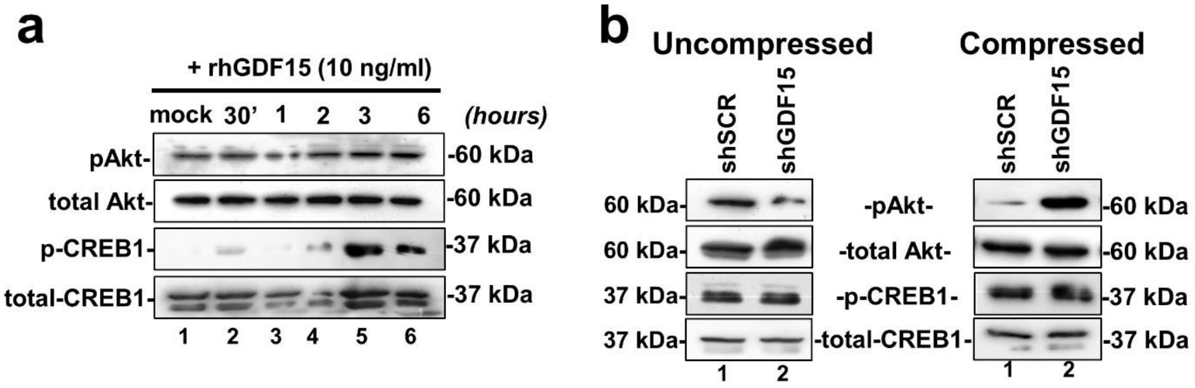

Supplementary Figure 6. Full length western blots.

a.

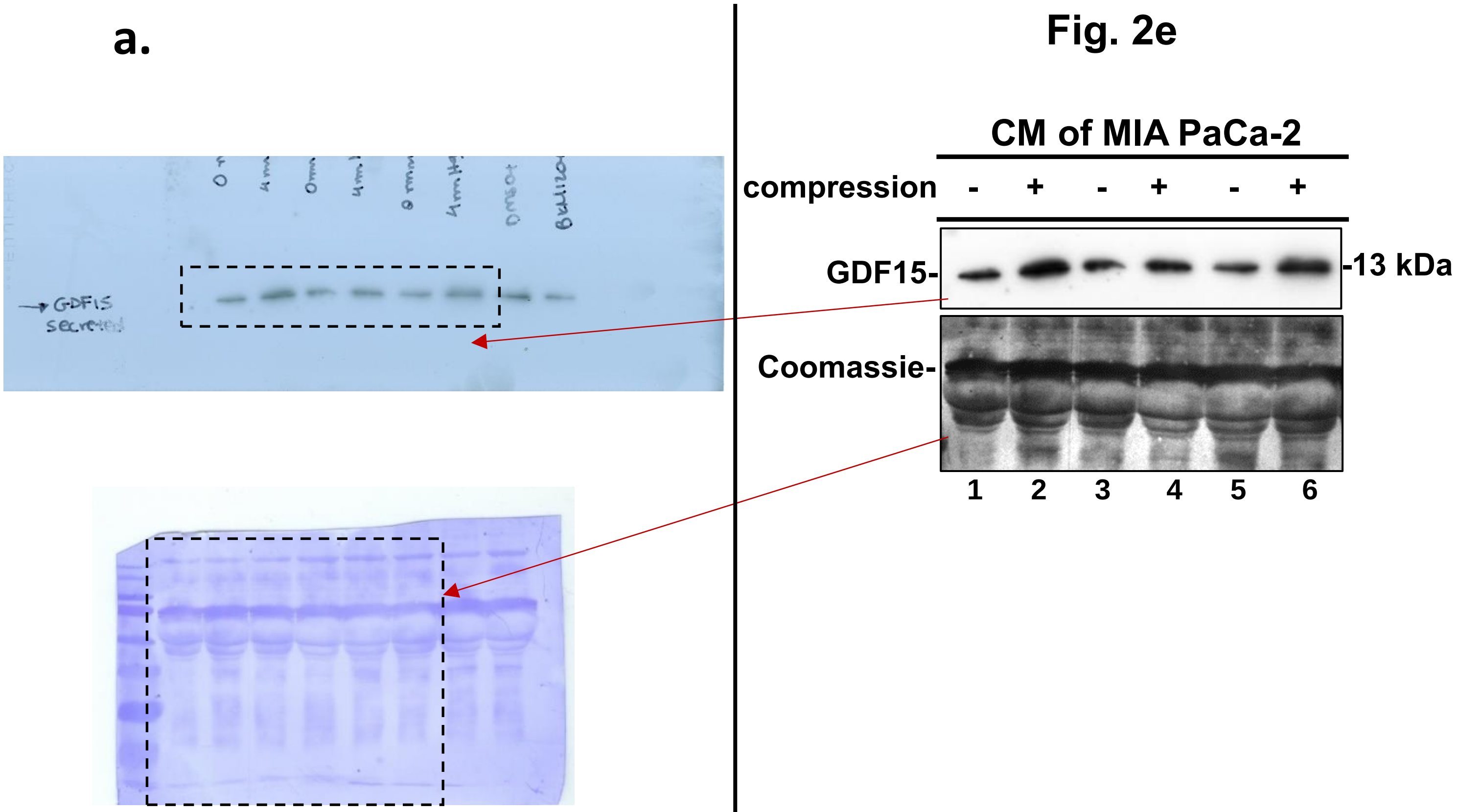

b.

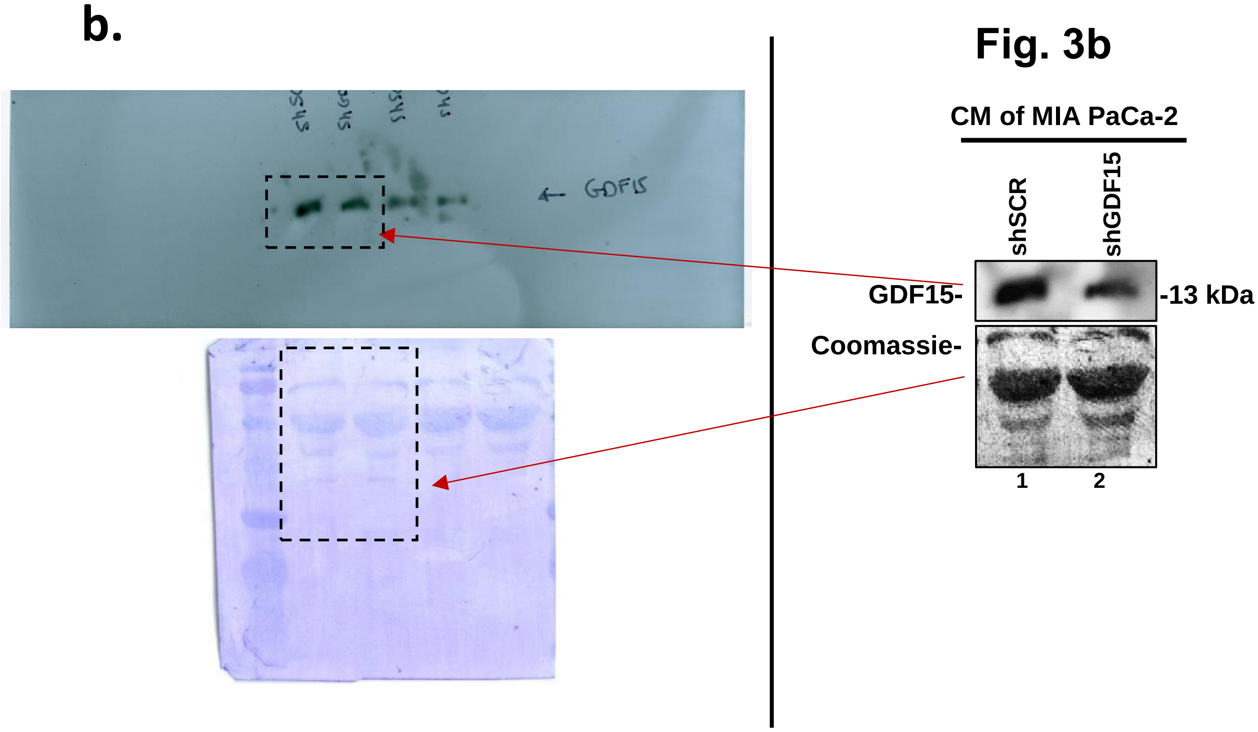

c.

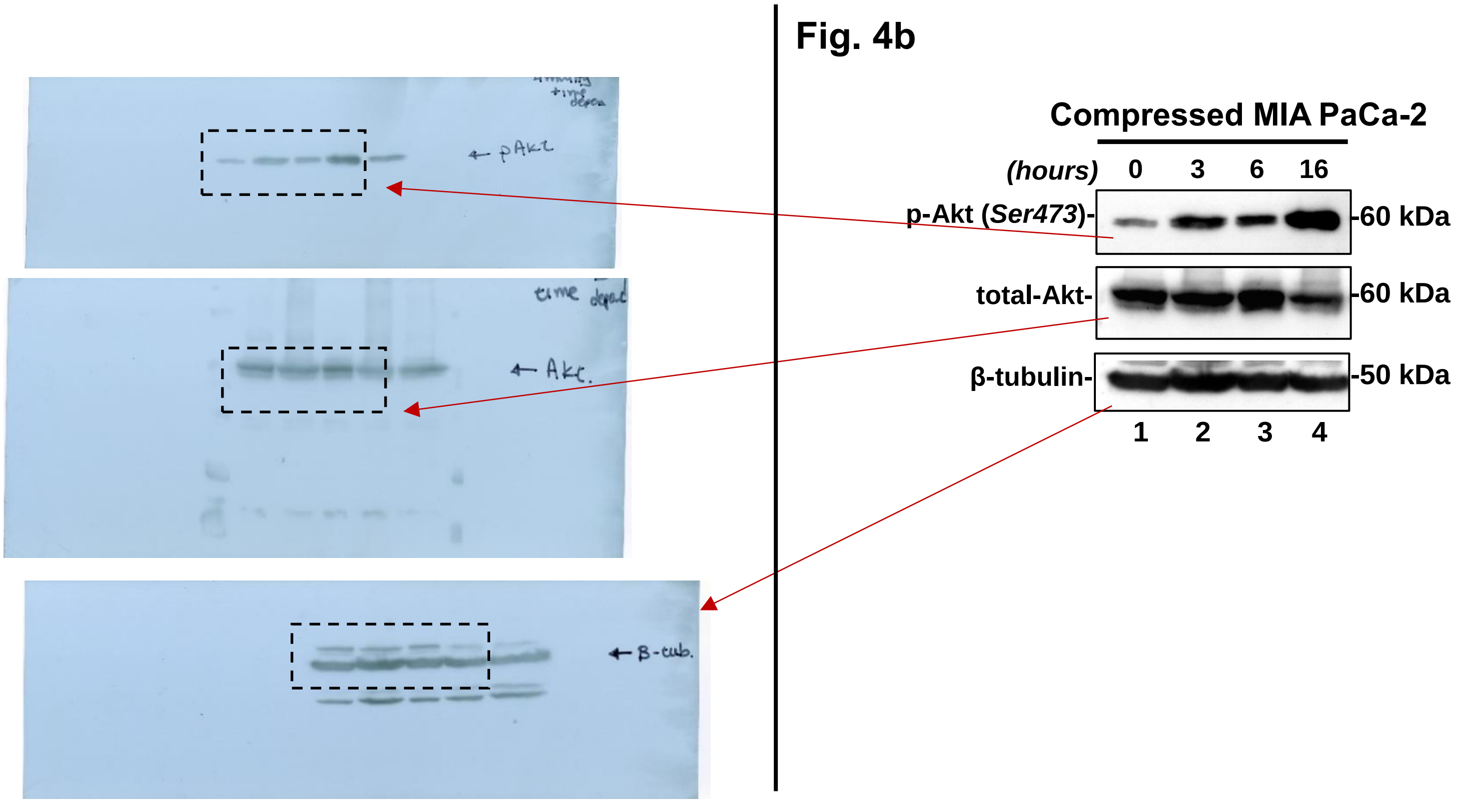

← pAkt
time depd
← Akt.
← β-tub.
Fig. 4b
Compressed MIA PaCa-2
(hours) 0 3 6 16
p-Akt (Ser473)-
-60 kDa
total-Akt-
-60 kDa
β-tubulin-
-50 kDa
1 2 3 4

d.

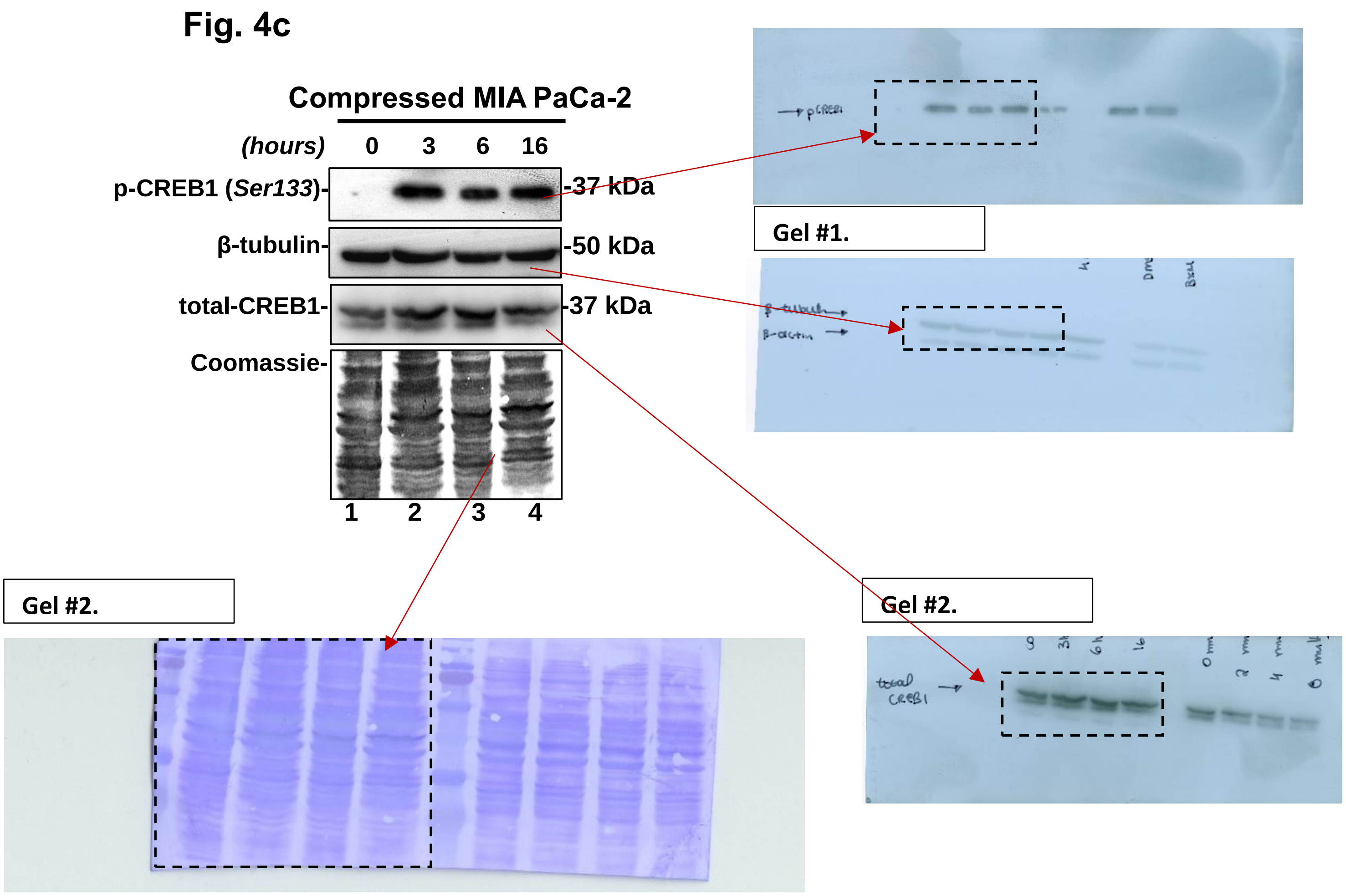
Fig. 4c
Compressed MIA PaCa-2
(hours) 0 3 6 16
p-CREB1 (Ser133)-
-37 kDa
β-tubulin-
-50 kDa
total-CREB1-
-37 kDa
Coomassie-
1 2 3 4
Gel #1.
Gel #1.
Gel #2.
Gel #2.

e.

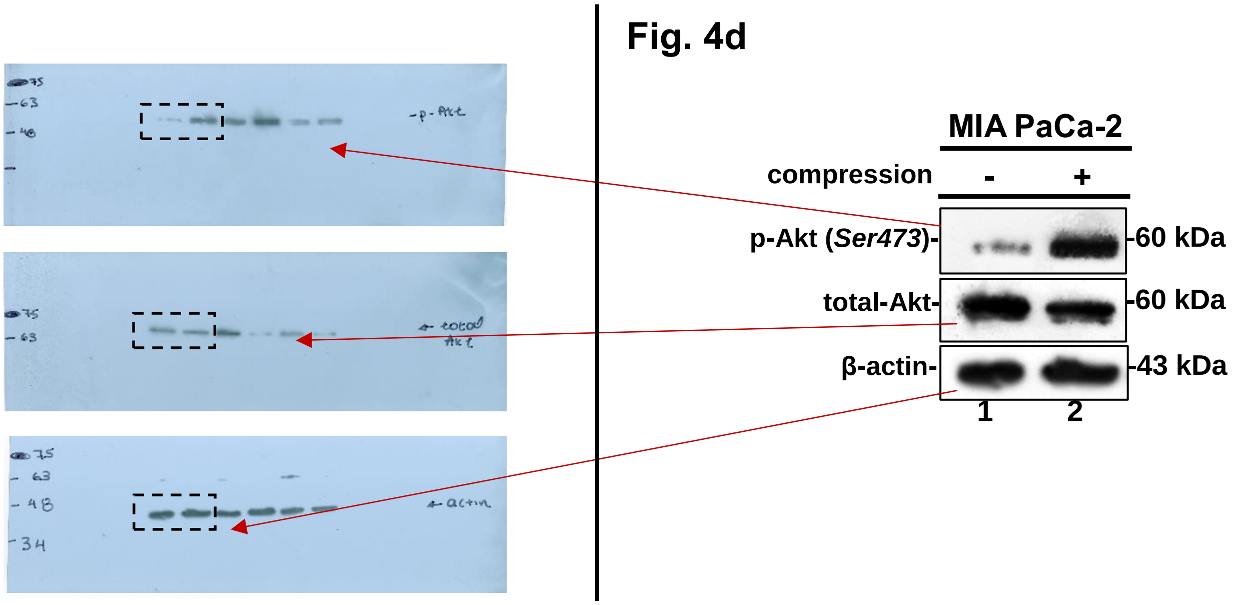
Fig. 4d
MIA PaCa-2
compression
-
+
p-Akt (*Ser473*)-
total-Akt-
β-actin-
-60 kDa
-60 kDa
-43 kDa
1
2

f.

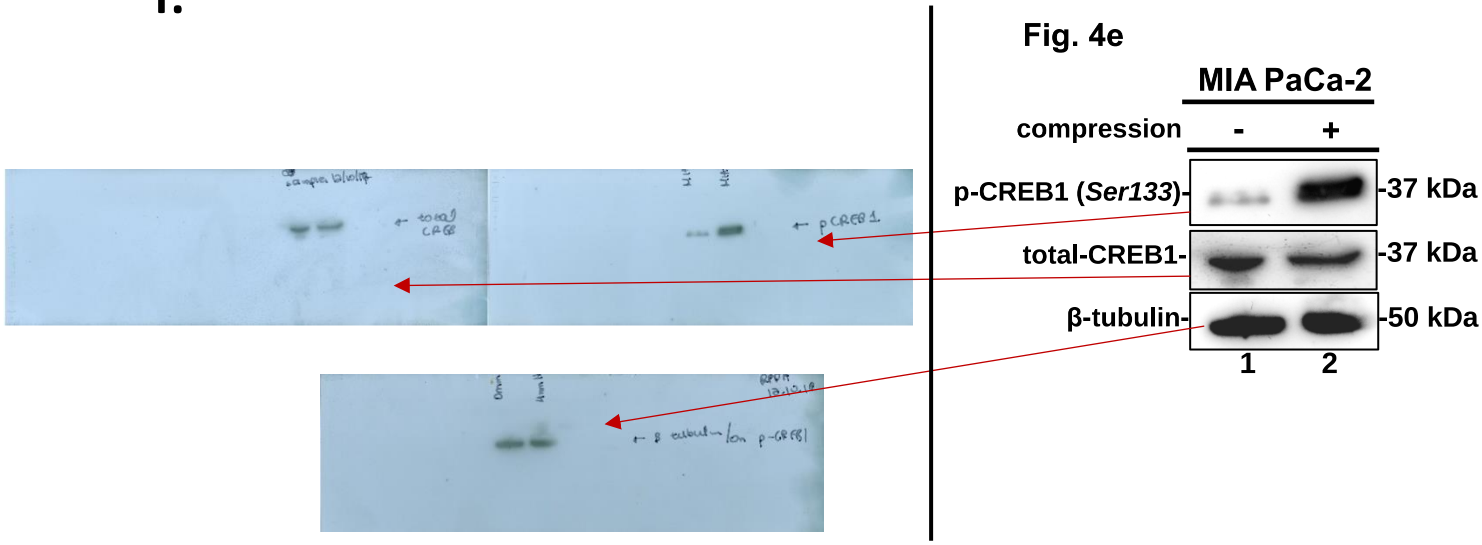
Fig. 4e
MIA PaCa-2
compression
-
+
p-CREB1 (*Ser133*)-
total-CREB1-
β-tubulin-
-37 kDa
-37 kDa
-50 kDa
1
2

g.

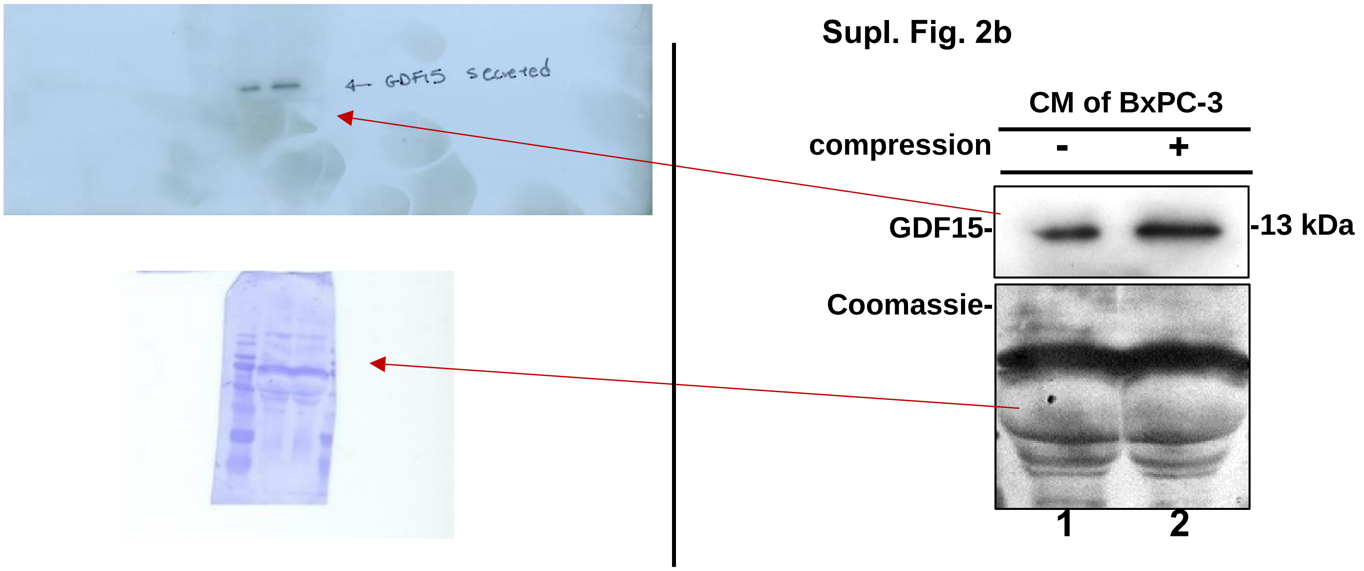
GDF15 secreted
Supl. Fig. 2b
CM of BxPC-3
compression
-
+
GDF15-
-13 kDa
Coomassie-
1
2

h.

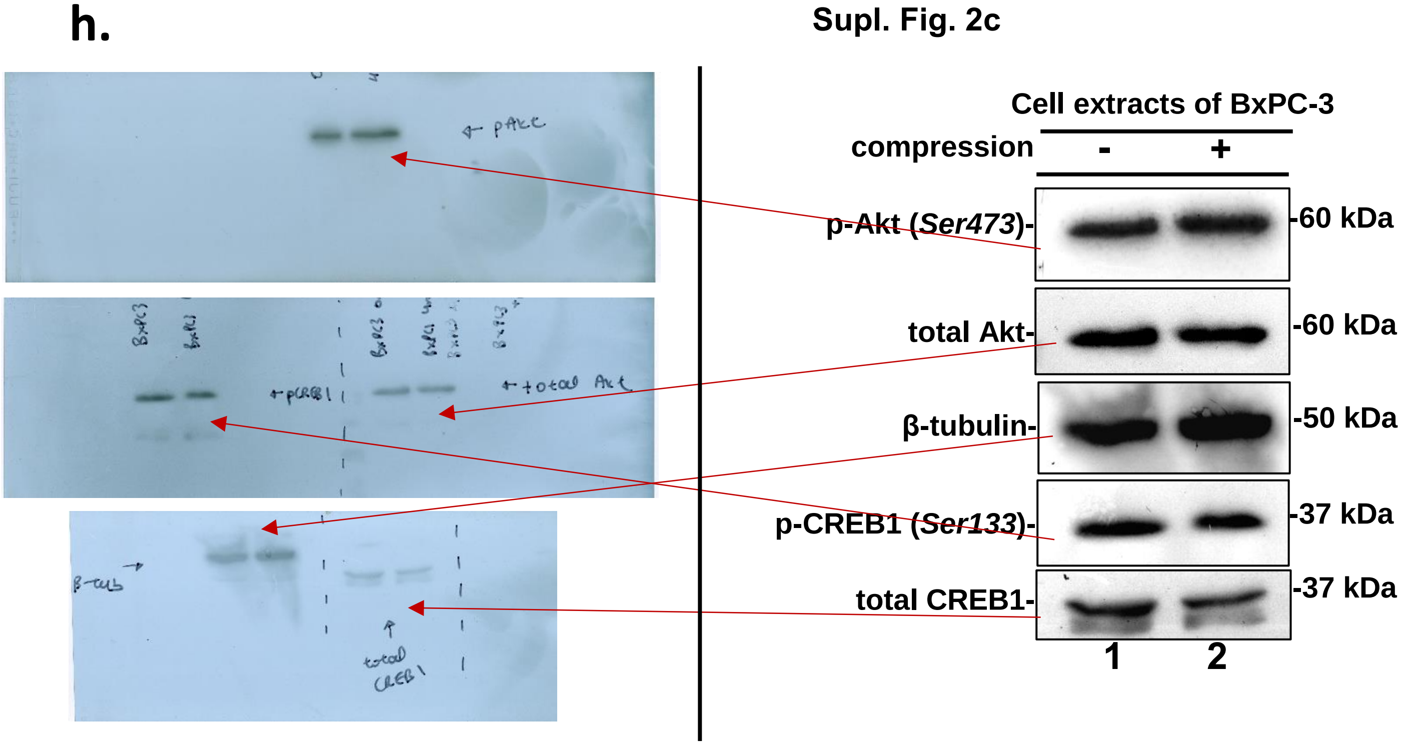
Supl. Fig. 2c
pAkt
pCREB1
total Akt
β-tub
total CREB1
Cell extracts of BxPC-3
compression
-
+
p-Akt (*Ser473*)-
-60 kDa
total Akt-
-60 kDa
β-tubulin-
-50 kDa
p-CREB1 (*Ser133*)-
-37 kDa
total CREB1-
-37 kDa
1
2

i.

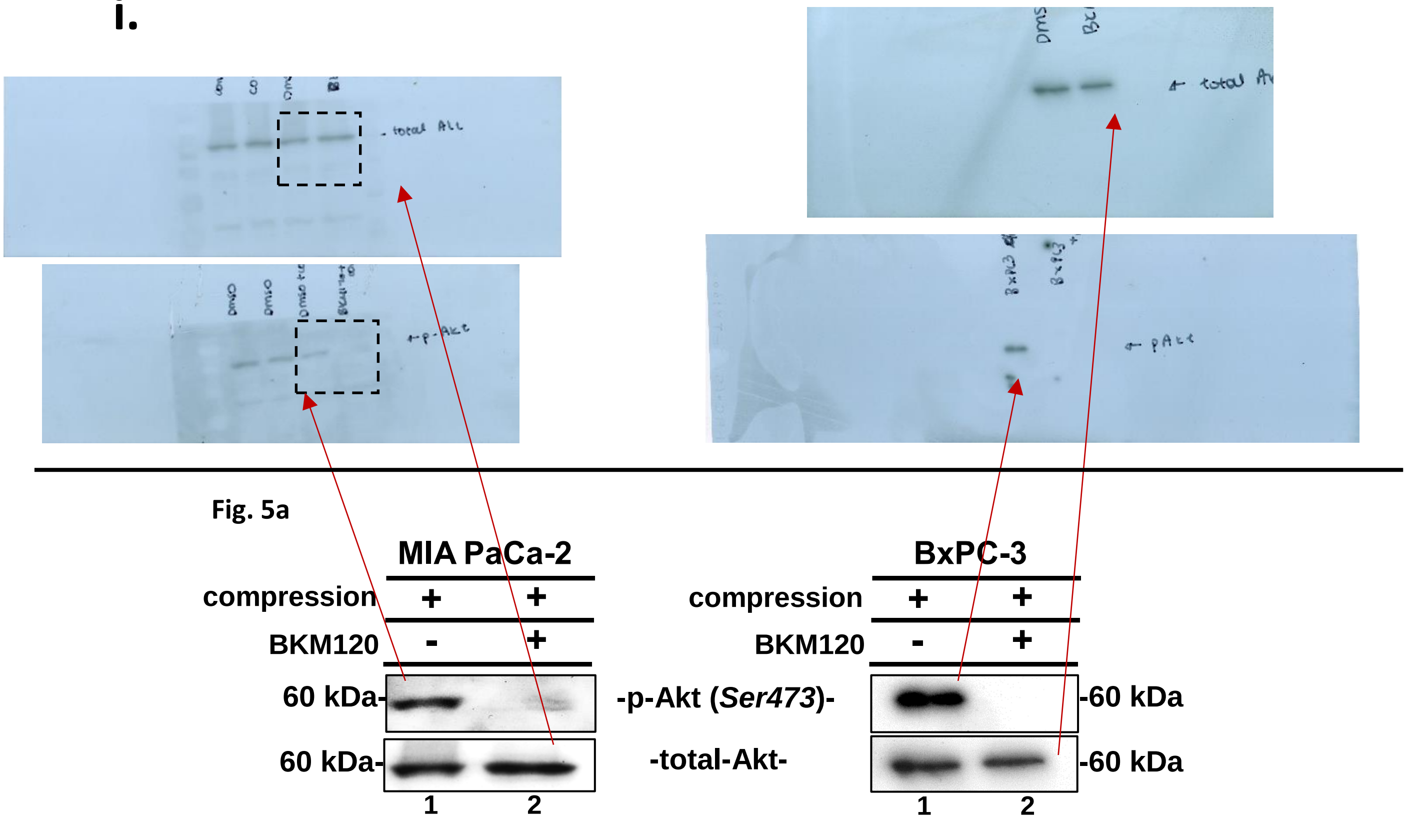
Fig. 5a
MIA PaCa-2
BxPC-3
compression
+
+
BKM120
-
+
60 kDa-
-p-Akt (Ser473)-
-60 kDa
60 kDa-
-total-Akt-
-60 kDa
1
2

j.

Fig. 6c

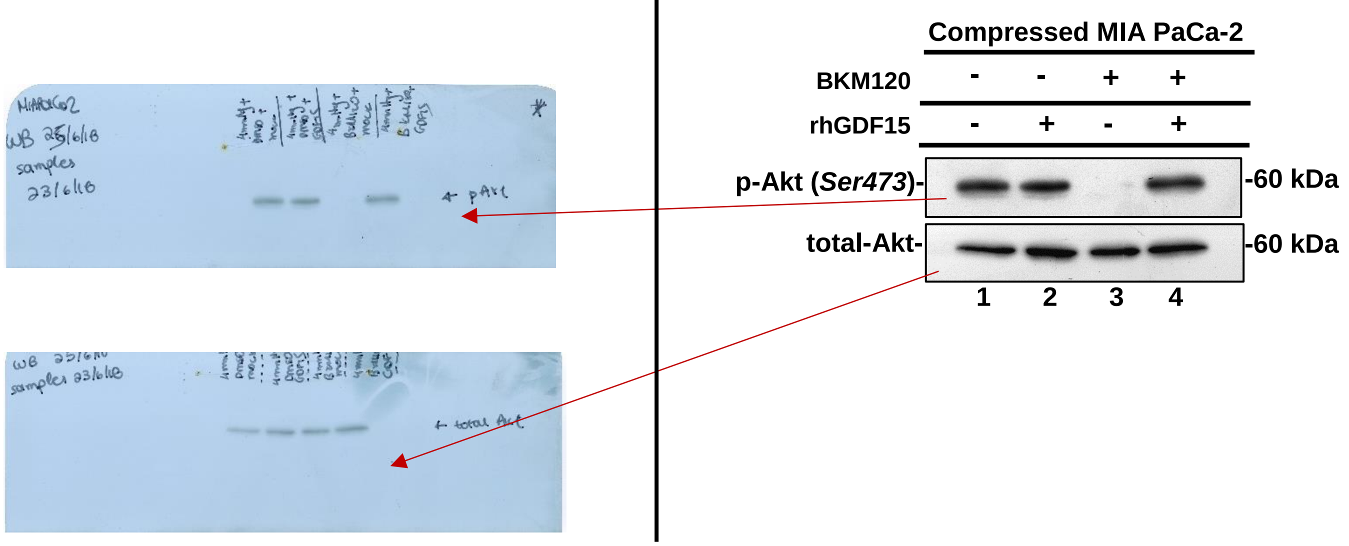

k.

Fig. 7a

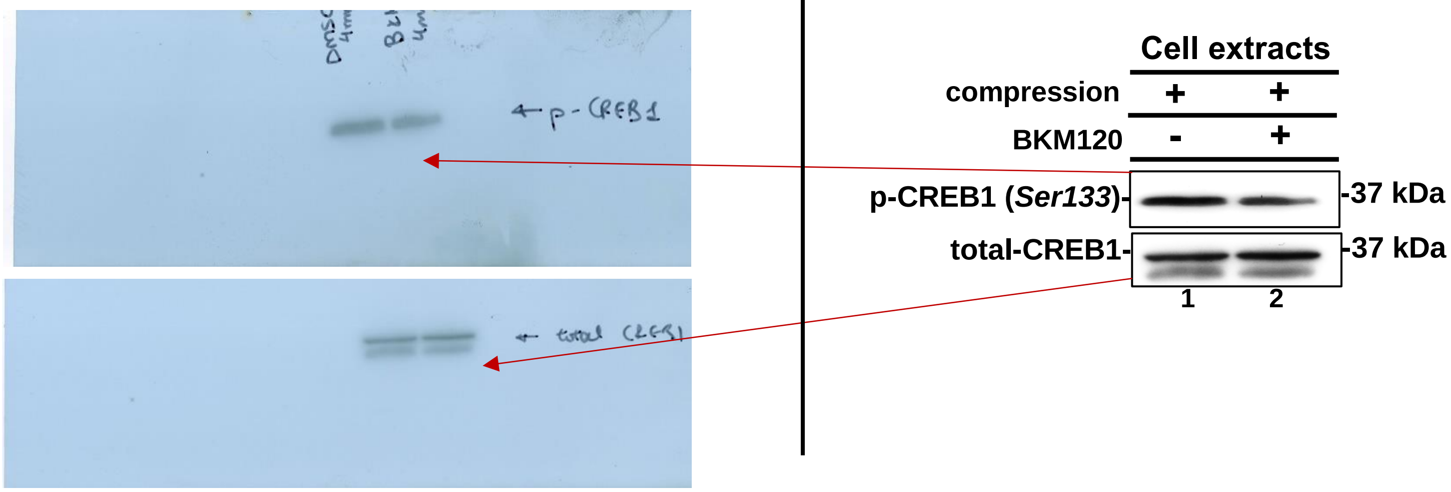

l.

Fig. 7d

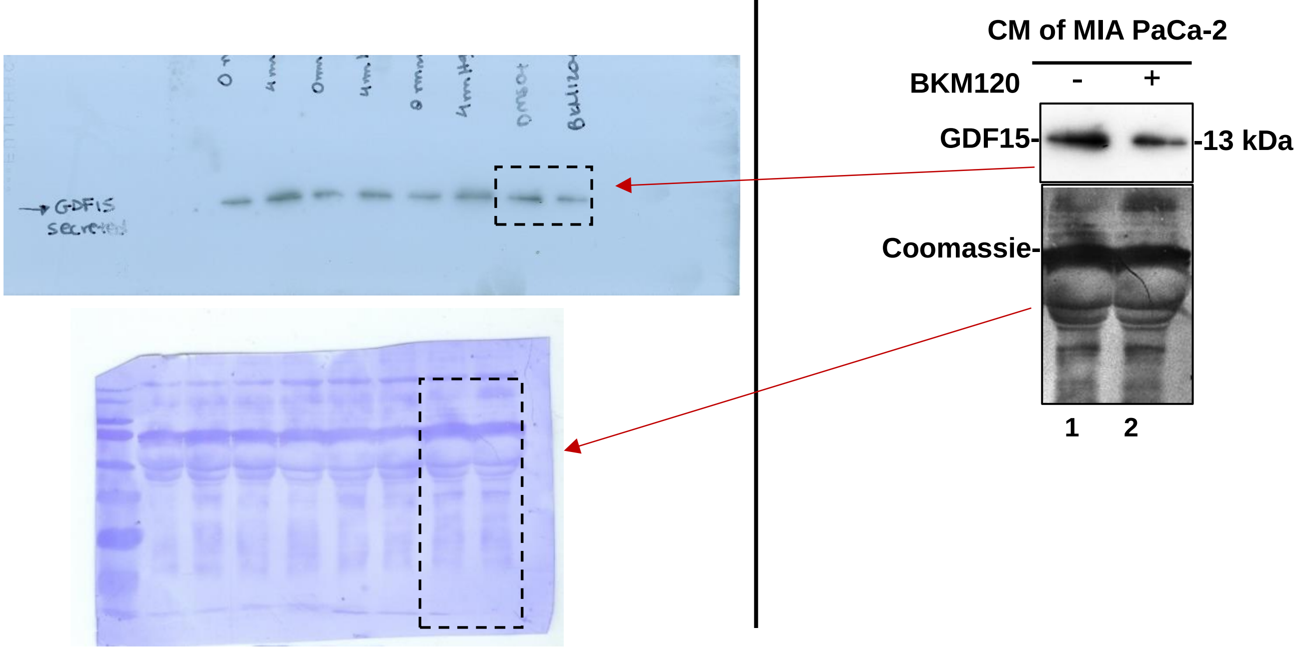

m.

Suppl.Fig. 4b

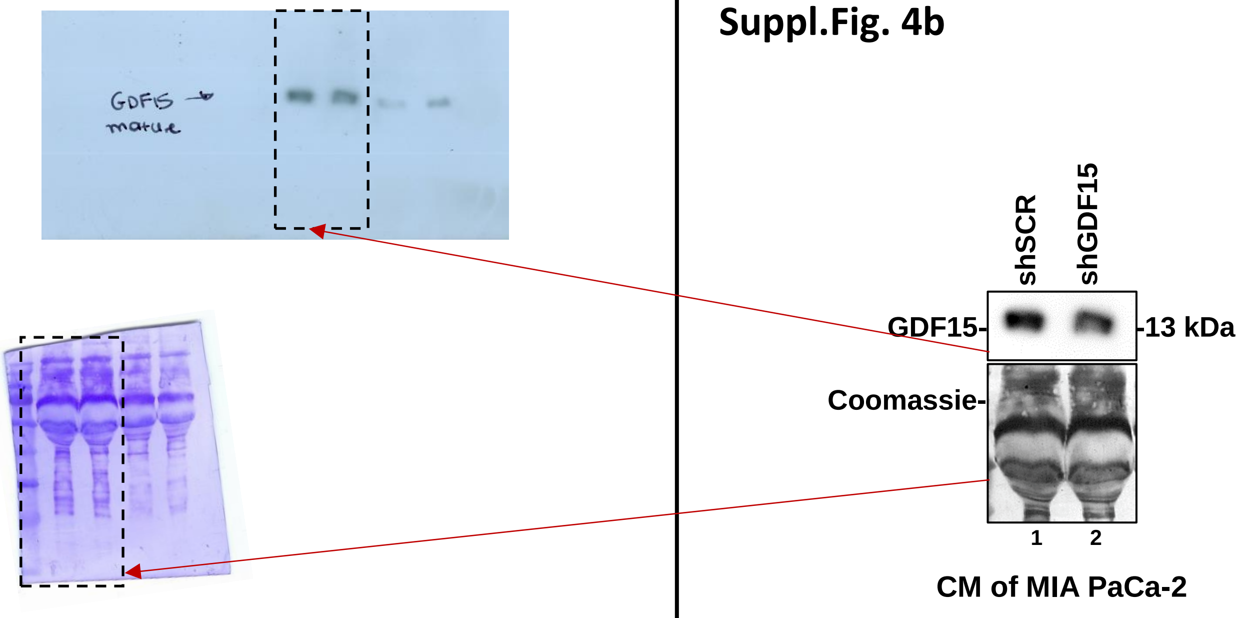

n.

Suppl.Fig. 4b

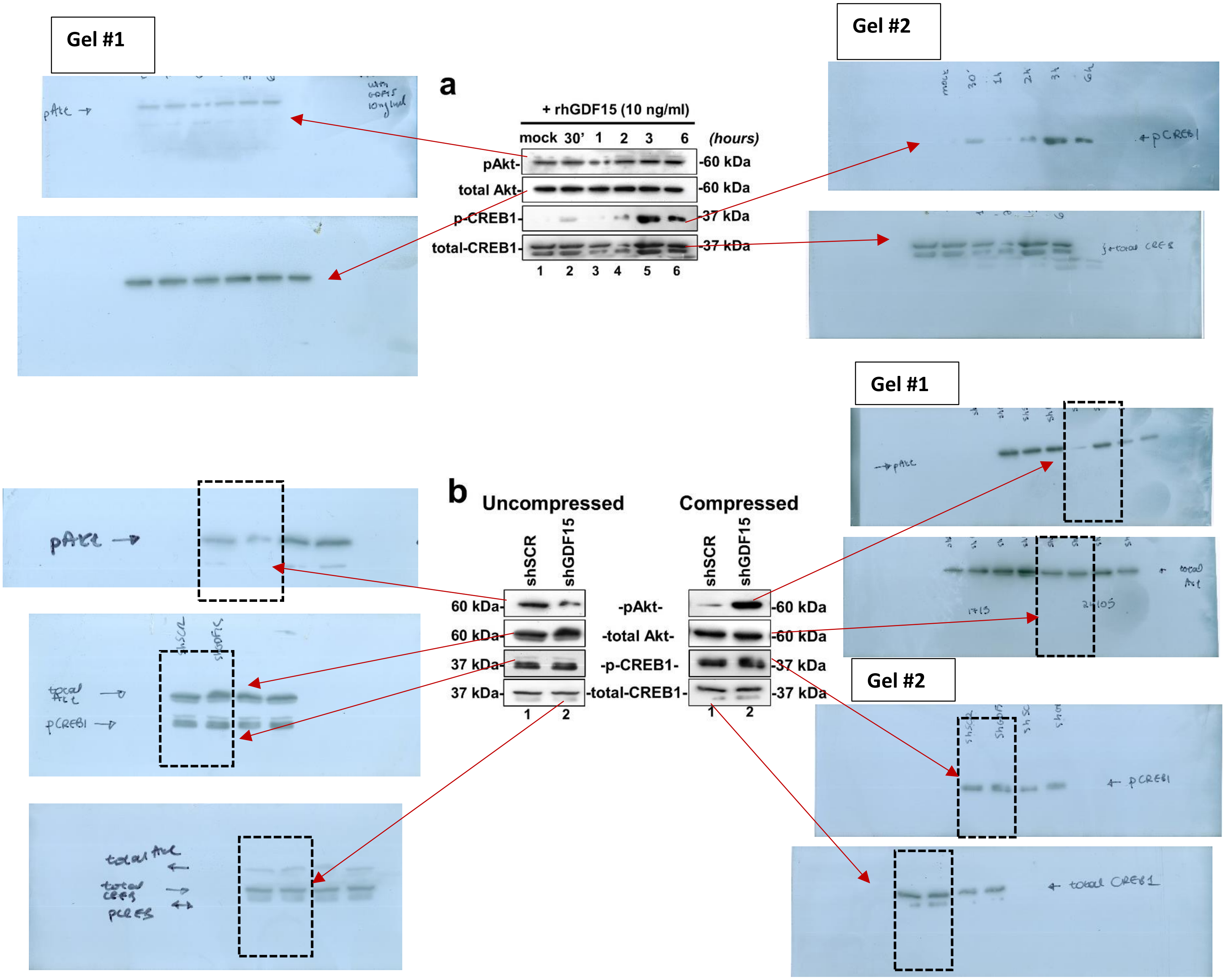

Supplementary Table 1. Primers used for qPCR

Primer Name	Primer sequence
β-actin	Forward: 5'-CGAGCACAGAGCCTCGCCTTTGCC-3'
	Reverse: 5'-TGTCGACGACGAGCGCGGCGATAT-3'
GDF15	Forward: 5'-TCAAGGTCGTGGGACGTGACA-3'
	Reverse: 5'- GCCGTGCGGACGAAGATTCT-3'
RhoA	Forward: 5'-CGGGAGCTAGCCAAGATGAAG-3'
	Reverse: 5'-CCTTGCAGAGCAGCTCTCGTA-3'
RhoB	Forward: 5'-TGCTGATCGTGTTCAGTAAG-3'
	Reverse: 5'-AGCACATGAGAATGACGTCG-3'
RhoC	Forward: 5'-TCCTCATCGTCTTCAGCAAG-3'
	Reverse: 5'-GAGGATGACATCAGTGTCCG-3'
